# Comprehensive characterization of multiple components of *Ziziphus jujuba Mill* using UHPLC‐Q‐Exactive Orbitrap Mass Spectrometers

**DOI:** 10.1002/fsn3.3020

**Published:** 2022-08-09

**Authors:** Shi‐han Qin, Fang Yan, Shuai E, Pei Xiong, Su‐nv Tang, Kai‐quan Yu, Min Zhang, Yung‐chi Cheng, Wei Cai

**Affiliations:** ^1^ School of Pharmacy Weifang Medical University Weifang China; ^2^ School of Pharmaceutical Sciences Hunan University of Medicine Huaihua China; ^3^ Department of Pharmacology Yale University School of Medicine New Haven Connecticut USA

**Keywords:** chemical components, parallel reaction monitoring scanning, UHPLC‐Q‐Exactive Orbitrap MS, *Ziziphus jujuba Mill*

## Abstract

*Ziziphus jujuba Mill* is the dried ripe fruit of the Rhamnaceae family; it is widely distributed in Shandong, Henan, Liaoning, and other places in China. In folk medicine, it was used to restore vital energy, as a blood tonic, and for the treatment of spleen deficiency. To date, a complete investigation of the compounds of *Z. jujuba* has rarely been performed. Therefore, a reliable strategy based on UHPLC‐Q‐Exactive Orbitrap MS, combined with trace data acquisition mode (parallel reaction monitoring scanning, PRM) and multiple data processing methods, is necessary for the characterization of compounds in the *Z. jujuba.* Ultimately, 295 compounds, including 69 flavonoids, 60 alkaloids, 82 phenylpropanoids, 52 organic acids, and 32 other components, were identified in the *Z. jujuba*; of these, 270 have been reported in *Z. jujuba* for the first time. This study provides deep insights into the chemistry of *Z. jujuba* and could be useful for further studies aimed at identifying the factors contributing to the health benefits attributed to this fruit.

## Graphical abstract

In this study, a reliable strategy based on UHPLC‐Q‐Exactive Orbitrap MS combined parallel reaction monitoring scanning mode was established to characterize the chemistry in *Z. Jujuba*. Eventually, 295 compounds were identified in *Z. jujuba*; of these, 270 have been reported in *Z. jujuba* for the first time

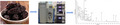

## 1 INTRODUCTION

*Ziziphus jujuba Mill* is the dried ripe fruit of the Rhamnaceae family; it is widely distributed in Shandong, Henan, Liaoning, and other places in China (Jiang, 2019; Yang, 2020). *Z. jujuba* has a long history of use for nutritive purposes and the treatment of a broad‐spectrum of diseases. The health benefits of *Z. jujuba* include immunomodulatory, antitumor, antioxidant, hepatoprotective, and hypoglycemic activities and gastrointestinal–protective effects (Hong et al., 2020; Ji et al., 2020; Yu et al., 2016). Additionally, it can also prolong the life span by nourishing the blood and improving sleep quality. Due to its good pharmacological effect and taste, *Z. jujuba* is extensively used in food and pharmaceutical industries.

The greatest nutritional value of *Z. jujuba* is its rich dietary fiber and fructose content, which may regulate blood sugar levels by slowing digestion (Li et al., 2007) and control calorie intake through its satiating effect (Gao et al., 2012). In addition, *Z. jujuba* possesses a large number of vitamins and minerals, which can enhance immunity in the body (Zhang, 2002). At present, *Z. jujuba* is processed into various widely consumed foods such as date dumplings, candies, and wine (Han et al., 2021). Hence, the chemical composition of *Z. jujuba* is worth studying to provide basis for the development and utilization of functional foods and pharmaceuticals.

In recent years, liquid chromatography mass spectrometry (LC–MS) is increasingly being used to identify the chemical components and metabolites of various plant medicines (Qin et al., 2021; Xiong et al., 2021). It shows a wide range of analysis and strong separation ability and yields reliable qualitative analysis results (Liang et al., 2022). In this study, the UHPLC‐Q‐Exactive Orbitrap MS combined with PRM was adopted to characterize the compounds in *Z. jujuba* (Gong et al., 2020; Qin et al., 2021). Finally, 295 compounds were identified in *Z. jujuba*. Among these, 270 were reported for the first time. The present study aimed to provide relatively comprehensive information about the main compounds in *Z. jujuba* to enhance its value.

## 2 EXPERIMENTAL STUDY

### 2.1 Materials and chemicals

Catechin, quercetin, luteolin 7‐O‐glucoside, quercitrin, hyperoside, isoquercitrin, sinomenine, kaempferol‐3‐O‐rutinoside, kaempferol‐3‐O‐neohesperidoside, and quercetin 3‐O‐rutinoside were purchased from the Chengdu Refine Biology Co., Ltd. (Sichuan, China). Caffeic acid, magnoflorine, phlorizin, procyanidin B2, and proanthocyanidins were provided by Sichuan Weikeqi Biotechnology Co., Ltd. (Sichuan, China). Quinic acid, oleanic acid (OA), cyclic adenosine monophosphate (cAMP), cyclic guanosine monophosphate (CGMP), trilobatin, and ferulic acid were acquired by Chengdu Purechem‐Standard Co., Ltd. (Sichuan, China). The purity of the standard compounds was no less than 98% based on LC‐UV. *Z. jujuba* was provided by Sun Ten Pharmaceutical Co., Ltd. (Taiwan, China).

The HPLC‐grade acetonitrile and methanol were purchased from Mackin company. The MS grade formic acid was received from the Thermo Fisher Scientific Co., Ltd. (USA). The deionized water was purchased from Watsons Water (Guangzhou, China). Other solvents were of an analytical grade.

### 2.2 Standard and sample preparation

*Ziziphus jujuba* (1 g) was accurately weighed and sonication with 20 ml of methanol/deionized water (7:3) was performed for 1 hour; the extracting solution was centrifuged (10 min, 10°C, 13523 *g*) to obtain the supernatant and dried under the stream of nitrogen at room temperature to obtain the residues, which were reconstituted in 0.1 ml of methanol. Finally, the supernatant was added to the LC–MS for further analysis.

The 21 reference standards were accurately weighed and dissolved in methanol to obtain reference standard solutions (0.1 mg/ml).

### 2.3 Instruments and conditions

An Dionex Ultimate 3000 UHPLC (Thermo Fisher Scientific, San Jose, CA, USA) and the Q‐Exactive Focus Orbitrap MS, equipped with an electrospray ionization (ESI) source, were used for acquiring the MS and MS^2^ data of *Z. jujuba*.

An Thermo Scientific Hypersil GOLD™ aQ (100 mm × 2.1 mm, 1.9 μm) was applied for chromatographic separation with a column temperature of 40°C with a flow rate of 0.3 ml/min. The mobile phase consisted of water containing 0.1% formic acid as eluent A and acetonitrile containing 0.1% formic acid as eluent B. The flow rate was set at a linear gradient as follows: 95% A at 0–2 min; 95–85% A at 2–5 min; 85–65% A at 5–20 min; 65–45% A at 20–30 min; 45–20% A at 30–40 min; 20–5% A at 40–45 min; 5–95% A at 45–45 min; and 95% A at 45–50 min. The sample injection volume was 2 μl.

MS analysis was running in both positive and negative ionization modes using an electrospray ionization (ESI). The key parameters were as follows: spray voltage, 3.5 kV (+); spray voltage, 3.2 kV (−); the sheath gas flow rate, 35 arb; aux gas flow rate, 10 arb; capillary temperature, 320°C; heater temperature, 350°C; S‐lens RF level, 60. The scan modes were full MS with a resolution of 70,000 and MS^2^ spectra were obtained by PRM triggered by inclusion ions list (Cai et al., 2020); the scan range was *m/z* 100–1500. The stepped, normalized collision energies were 20%, 40%, and 60%. Data acquisition and processing were carried out with the Xcalibur version 4.2 and Compound Discovery version 3.0 (Thermo Fisher Scientific, California, USA).

### 2.4 Expected compound prediction

In accordance with the understanding of the structure of compounds, the chemical constituents in the same category possess identical carbon skeletons and homologous biosynthetic pathways. For example, flavonoids possessed the carbon skeleton flavone and the type and number of substituent group including methyl (CH_3_), oxhydryl (OH), methoxyl (OCH_3_), glucose (C_6_H_10_O_5_, 162.0528), arabinose (C_5_H_8_O_4_, 132.0422), rhamnose (C_6_H_10_O_4_, 146.0579), rutinose (C_12_H_20_O_9_, 308.1107), lactose (C_12_H_20_O_10_, 324.1056), gentianose (C_18_H_30_O_15_, 486.1584), glucuronic acid (C_6_H_8_O_6_, 176.0320), and xyluronic acid (C_5_H_6_O_5_, 146.0215). Therefore, the molecule could be predicted based on this strategy (Cai et al., 2020).

## 3 RESULTS AND DISCUSSION

### 3.1 Analytical strategy

To screen and identify components systematically in *Z. jujub*a, an analytical strategy based on UHPLC‐Q‐Exactive Orbitrap MS with PRM was established in this study. Firstly, *Z. jujub*a was extracted and enriched by ultrasonic extraction with 70% methanol (20 ml). Secondly, the sample was injected into UHPLC‐Q‐Exactive Orbitrap MS to gain high‐resolution MS data for the trace components in *Z. jujub*a; the MS^2^ data can be obtained by the PRM mode triggered by the inclusion ions list. Thirdly, the data of *Z. jujub*a were processed by Compound Discover version 3.0 modified metabolism workflow and expected compounds predicted. Finally, the components were identified based on standard fragmentation ions, retention time, and bibliography.

### 3.2 Optimization of UHPLC‐Q‐Exactive Orbitrap Mass Spectrometer

The raw data were captured on the UHPLC‐Q‐Exactive Orbitrap MS with the PRM acquisition strategy, which combines the characteristically fast scanning capability and MS^2^ data mining of trace components. The components were identified using Xcalibur version 4.2 and comparing the obtained precursor ions and corresponding fragment ions with the standard and documents.

This study investigated three collision energies: 40%; 60%, and stepped; and normalized collision energies 20%, 40%, and 60%. The results showed that, when the collision energy was 20%, 40%, and 60%, more suitable desugared or dehydrated fragment ions were generated, which was beneficial for detection. In addition, MS and MS/MS features, including flavonoids, phenylpropanoids, and organic acids, were quite abundant in negative ion mode, and alkaloids were found in positive ion mode. Therefore, we decided to choose two ion scan modes for this qualitative analysis. In addition, adding 0.1 formic acid to the mobile phase not only improves the chromatographic peak but also generates more adduct ion, which is helpful for the identification of compounds.

### 3.3 Characterization of the *Z. jujuba*

**FIGURE 1:**
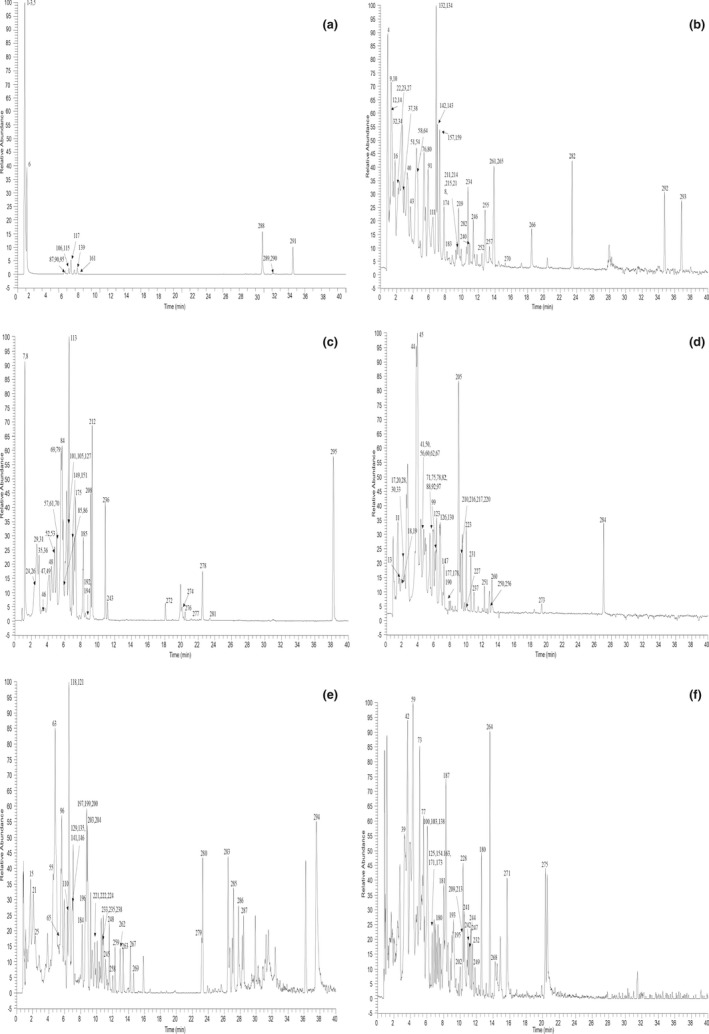
The high‐resolution extraction ion chromatography of *Z. jujuba* in negative ion mode a: 341.1089, 485.3272, 191.0197, 355.1035, 595.1663, 593.2087; b: 463.0882, 445.1140, 445.0776, 517.1351, 490.0981, 545.3484, 533.1512, 151.0401, 579.2083, 453.3010, 207.0299, 299.0772, 167.0350, 207.0663, 821.3965, 449.1089, 755.2040, 579.1719, 191.0561, 289.0718; c: 475.1457, 593.1723, 193.0506, 175.0612, 329.0878, 315.1085, 165.0552, 371.0984, 204.0666, 329.2334, 179.0350, 344.0402, 325.0929, 195.0299, 563.1618, 741.1884, 341.0878, 593.2087, 579.1355, 355.1035, 595.1663, 455.3531, 328.0452, 609.1461; d: 263.1283, 447.0933, 661.1774, 887.2463, 211.0248, 313.0929, 725.1935, 327.1085, 771.1989, 177.0193, 597.1825, 503.1406, 145.0506, 721.1985, 593.1512, 517.1563, 593.1301, 227.1289, 153.0193, 137.0244; e: 385.1657, 457.1351, 501.1402, 679.1879, 595.1305, 663.1935, 303.0510, 411.1297, 623.1618, 435.1297, 505.1351, 445.1352, 435.0933, 315.0722, 319.0818, 281.1391, 417.1191, 535.1974, 499.3065, 457.0988, 339.0722, 343.1035, 271.2273; f: 607.1305, 301.0354, 625.1410, 577.1351, 917.2568, 451.1246, 635.1981, 459.0933, 229.1445, 433.1140, 607.1668, 665.1935, 359.0984, 531.1508

**FIGURE 2:**
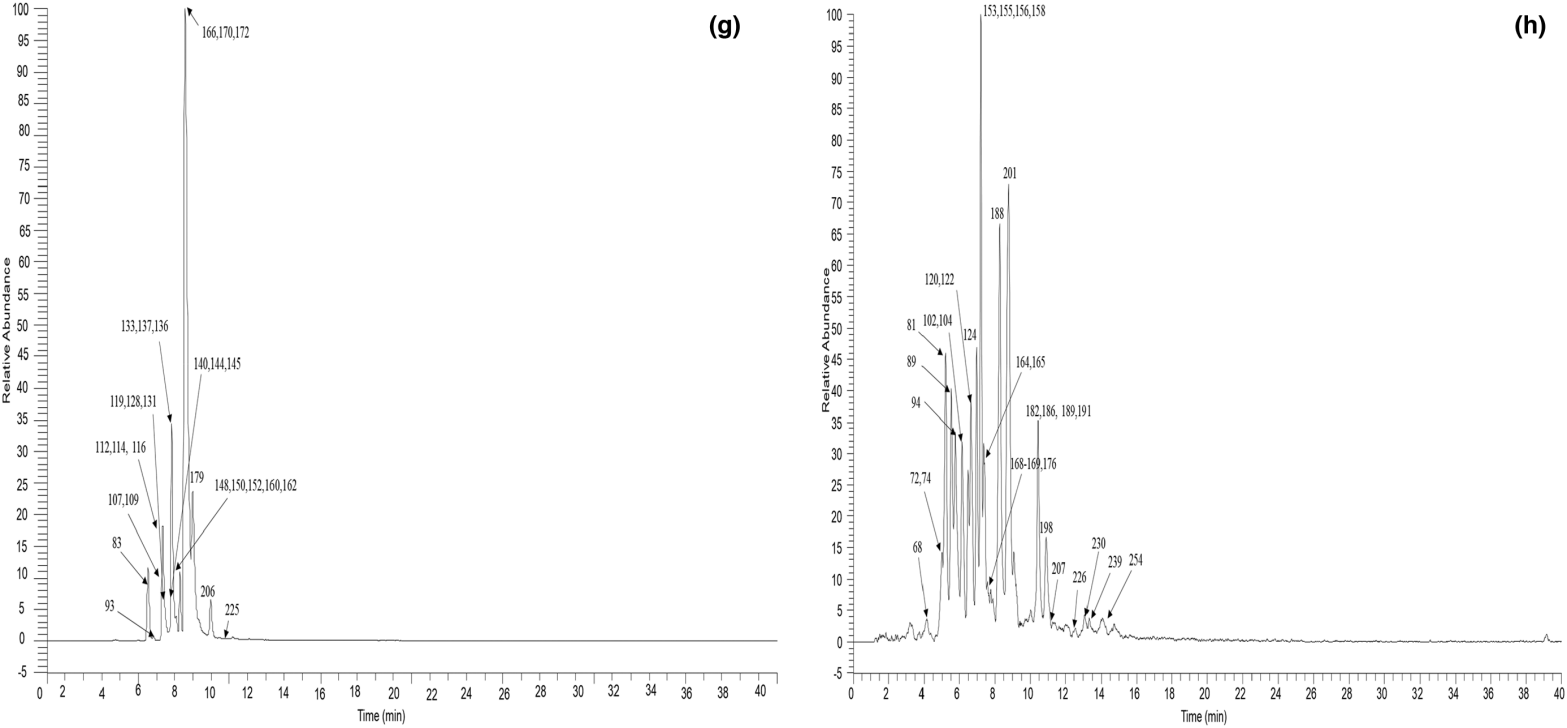
The high‐resolution extraction ion chromatography of *Z. jujuba* in positive ion mode g: 344.1862, 434.1809, 356.1498, 358.1654, 328.1543, 268.133, 448.1966, 314.1751, 314.1387, 286.1438, 342.1705; h: 358.2013, 296.1645, 400.1755, 356.1856, 312.1594, 272.1281, 344.1492, 326.1751, 282.1125, 302.1387, 374.1598, 316.1543, 330.1700, 340.1543, 300.1594

A total of 295 compounds (69 flavonoids, 60 alkaloids, 82 phenylpropanoids, 52 organic acids, and 32 other components) were identified in the *Z. jujuba* based on UHPLC‐Q‐Exactive Orbitrap MS combined with PRM, the mass spectrum information and retention time of compounds (Table 1). Figures 1 and 2 show the extracted ion chromatogram (EIC) in negative and positive ion modes.

**TABLE 1:** The chromatographic and mass data of detected components from *Z. jujuba* though UHPLC‐Q‐Exactive Orbitrap MS

Peak	*t*_*R*_	Theoretical mass *m/z*	Experimental mass *m/z*	Error (ppm)	Formula	MS/MS fragment (−)	MS/MS fragment (+)	Identification	Categories
1.	0.81	341.1089	341.1082	−2.30	C_12_H_22_O_11_	MS^2^[341]: 179.0549(100), 161.0443(30),143.0336(26)		Sucrose isomer	Other components
2.	0.86	191.0197	191.0185	−6.37	C_6_H_8_O_7_	MS^2^[191]: 111.0073(100)		Citric acid isomer	Organic acid
3.	0.89	341.1089	341.1083	−2.01	C_12_H_22_O_11_	MS^2^[341]: 179.0549(100), 161.0440(25), 143.0335(20)		Sucrose isomer	Other components
4.	0.89^a^	191.0561	191.0550	−5.87	C_7_H_12_O_6_	MS^2^[191]: 111.0073(100), 87.0072(50), 85.0280(30)		Quinic acid	Organic acid
5.	1.08	341.1089	341.1084	−1.48	C_12_H_22_O_11_	MS^2^[341]: 179.0550(100), 161.0442(28), 143.0338(18)		Sucrose isomer	Other components
6.	1.14	191.0197	191.0186	−5.89	C_6_H_8_O_7_	MS^2^[191]: 111.0073(100)		Citric acid isomer	Organic acid
7.	1.20^a^	344.0402	344.0401	−0.17	C_10_H_12_O_7_N_5_P	MS^2^[344]:150.0412(100), 78.9577(70), 133.0145(59)		Cyclic guanosine monophosphate	Other components
8.	1.20^a^	328.0452	328.0453	0.05	C_10_H_12_O_6_N_5_P	MS^2^[328]:134.0461(100), 78.9577(34)		Adenosine Cyclophosphate	Other components
9.	1.30	191.0561	191.0551	−5.24	C_7_H_12_O_6_	MS^2^[191]: 111.0073(100), 87.0073(49), 85.0280(29)		Quinic acid isomer	Organic acid
10.	1.37	490.0981	490.0979	0.96	C_16_H_22_O_11_N_5_P	MS^2^[490]: 176.0566(100), 78.9576(59), 134.0459(25), 328.0432(10)		Adenosine Cyclophosphate‐O‐glucoside	Other components
11.	1.41	145.0506	145.0496	−6.98	C_6_H_10_O_4_	MS^2^[145]:57.0333(100), 127.0389(51), 101.0595(40), 83.0489(40), 71.0489(31), 99.0439(25)		3‐Methylglutaric acid isomer	Organic acid
12.	1.43	191.0561	191.0551	−5.14	C_7_H_12_O_6_	MS^2^[191]: 87.0073(100), 111.0073(93), 85.0280(31)		Quinic acid isomer	Organic acid
13.	1.57	145.0506	145.0494	−8.77	C_6_H_10_O_4_	MS^2^[145]: 101.0593(100), 127.0387(87), 83.0487(76), 57.0331(76), 71.0487(59)		3‐Methylglutaric acid isomer	Organic acid
14.	1.59	151.0401	151.0388	−8.26	C_8_H_8_O_3_	MS^2^[151]: 123.0437(100), 108.0202(7), 136.0152(7)		Vanillin isomer	Organic acid
15.	1.75	315.0722	315.0719	−0.81	C_13_H_16_O_9_	MS^2^[315]: 153.0181(100), 109.0280(85), 123.0439(17), 108.0203(4)		Dihydroxybenzoic acid‐O‐glucoside	Organic acid
16.	1.80	299.0772	299.0769	−1.14	C_13_H_16_O_8_	MS^2^[299]: 93.0331(100), 137.0230(70)		4‐Hydroxybenzoic acid β‐glucoside	Organic acid
17.	1.95	153.0193	153.0181	−7.86	C_7_H_6_O_4_	MS^2^[153]: 109.0279(100), 81.0330(39), 123.0436(24)		Dihydroxybenzoic acid	Organic acid
18.	2.08	211.0248	211.0240	−3.99	C_9_H_8_O_6_	MS^2^[211]: 167.0338(96), 123.0438(34)		Vanillic acid derivative	Organic acid
19.	2.28	211.0248	211.0240	−3.99	C_9_H_8_O_6_	MS^2^[211]: 93.0331(100), 167.0337(89), 123.0438(81)		Vanillic acid derivative	Organic acid
20.	2.10	153.0193	153.0181	−8.05	C_7_H_6_O_4_	MS^2^[153]: 109.0282(100), 123.0435(58), 81.0330(24)		Dihydroxybenzoic acid	Organic acid
21.	2.12	435.0933	435.0927	−1.32	C_20_H_20_O_11_	MS^2^[435]: 137.0233(100), 109.0282(85), 289.0716(83)		Catechin‐O‐xyluronate	Flavonoid
22.	2.18	167.0350	167.0339	−6.72	C_8_H_8_O_4_	MS^2^[167]: 123.0438(100), 152.0103(24), 108.0202(16)		Vanillic acid isomer	Organic acid
23.	2.27	167.0350	167.0338	−7.02	C_8_H_8_O_4_	MS^2^[167]:152.0102(92), 108.0202(64), 123.0437(54)		Vanillic acid isomer	Organic acid
24.	2.34	329.0878	329.0875	−0.87	C_14_H_18_O_9_	MS^2^[329]: 167.0338(100), 108.0202(86), 152.0102(62), 123.0437(36)		Vanillic acid‐O‐glucoside	Organic acid
25.	2.35	315.0722	315.0771	−0.64	C_13_H_16_O_9_	MS^2^[315]: 123.0438(100), 153.0188(6), 109.0281(5)		Dihydroxybenzoic acid‐O‐glucoside	Organic acid
26.	2.37	315.1085	315.1084	−0.35	C_14_H_20_O_8_	MS^2^[315]: 153.0544(100), 123.0437(90)		Hydroxytyrosol‐4‐glucoside	Organic acid
27.	2.38	299.0772	299.0770	−0.91	C_13_H_16_O_8_	MS^2^[299]: 137.0230(100), 93.0331(5)		4‐Hydroxybenzoic acid β‐glucoside	Organic acid
28.	2.47	153.0193	153.0181	−8.25	C_7_H_6_O_4_	MS^2^[153]: 109.0281(100), 108.0203(7), 123.0438(5), 81.0331(2)		Dihydroxybenzoic acid	Organic acid
29.	2.59	329.0878	329.0874	−1.14	C_14_H_18_O_9_	MS^2^[329]: 167.0338(100), 108.0203(86), 152.0103(58), 123.0438(37)		Vanillic acid‐O‐glucoside	Organic acid
30.	2.62	153.0193	153.0181	−8.18	C_7_H_6_O_4_	MS^2^[153]: 109.0281(100), 108.0203(6), 123.0438(5), 81.0331(2)		Dihydroxybenzoic acid	Organic acid
31.	2.64	315.1085	315.1084	−0.45	C_14_H_20_O_8_	MS^2^[315]: 153.0545(100), 123.0438(89), 95.0488(1)		Hydroxytyrosol‐4‐glucoside	Organic acid
32.	2.66	299.0772	299.0768	−1.34	C_13_H_16_O_8_	MS^2^[299]: 137.0231(100), 93.0331(9)		4‐Hydroxybenzoic acid β‐glucoside	Organic acid
33.	2.71	503.1406	503.1403	−0.61	C_21_H_28_O_14_	MS^2^[503]: 161.0232(100), 341.0872(33), 179.0340(19)		Caffeic acid‐O‐lactoside	Phenylpropanoid
34.	2.71	533.1512	533.1509	−0.59	C_22_H_30_O_15_	MS^2^[533]: 145.0282(100), 325.0921(71), 163.0387(67), 119.0490(66)		P‐Coumaric acid derivative	Phenylpropanoid
35.	2.71	195.0299	195.0289	−5.01	C_9_H_8_O_5_	MS^2^[195]: 123.0437(100), 151.0388(61), 136.0153(11)		Vanillin derivative	Organic acid
36.	2.91	195.0299	195.0289	−5.11	C_9_H_8_O_5_	MS^2^[195]: 123.0437(100), 151.0388(57), 136.0153(12)		Vanillin derivative	Organic acid
37.	3.01	299.0772	299.0768	−1.44	C_13_H_16_O_8_	MS^2^[299]: 137.0231(100), 93.0332(10)		4‐Hydroxybenzoic acid β‐glucoside	Organic acid
38.	3.27	299.0772	299.0768	−1.34	C_13_H_16_O_8_	MS^2^[299]: 137.0230(100)		4‐Hydroxybenzoic acid β‐glucoside	Organic acid
39.	3.29	359.0984	359.0981	−0.72	C_15_H_20_O_10_	MS^2^[359]: 138.0312(100), 182.0213(82), 197.0448(48), 153.0548(41)		Syringic acid‐O‐glucoside	Organic acid
40.	3.38	533.1512	533.1507	−0.93	C_22_H_30_O_15_	MS^2^[533]: 265.0716(100), 163.0388(68), 119.0488(59), 145.0283(59)		P‐Coumaric acid derivative	Phenylpropanoid
41.	3.56	503.1406	503.1401	−0.97	C_21_H_28_O_14_	MS^2^[503]: 179.0338(100), 161.0232(86), 135.0438(83), 281.0661(66), 221.0447(48)		Caffeic acid‐O‐lactoside	Phenylpropanoid
42.	3.65	359.0984	359.0980	−0.98	C_15_H_20_O_10_	MS^2^[359]: 138.0309(100), 197.0445(97), 182.0208(60), 153.0536(29)		Syringic acid‐O‐glucoside	Organic acid
43.	3.69	533.1512	533.1507	−0.93	C_22_H_30_O_15_	MS^2^[533]: 163.0388(100), 119.0488(92), 325.0926(30), 145.0284(18)		P‐Coumaric acid derivative	Phenylpropanoid
44.	3.72	137.0244	137.0231	−9.61	C_7_H_6_O_3_	MS^2^[137]: 93.0331(100)		4‐Hydroxybenzoic acid isomer	Organic acid
45.	3.86	137.0244	137.0231	−9.47	C_7_H_6_O_3_	MS^2^[137]: 93.0331(100)		4‐Hydroxybenzoic acid isomer	Organic acid
46.	3.97	341.0878	341.0874	−1.19	C_15_H_18_O_9_	MS^2^[341]: 161.0232(100), 59.0124(37), 89.0230(22), 179.0338(14), 135.0438(11)		Caffeic acid‐O‐glucoside	Phenylpropanoid
47.	4.02	175.0612	175.0604	−4.84	C_7_H_12_O_5_	MS^2^[175]:115.0389(100), 85.0646(44), 113.0596(38)		2‐Isopropylmalic acid isomer	Organic acid
48.	4.10	563.1618	563.1613	−0.78	C_23_H_32_O_16_	MS^2^[563]: 193.0496(100), 175.0390(81), 355.1025(64), 134.0361(53), 160.0155(38)		Ferulic acid‐O‐derivative	Phenylpropanoid
49.	4.13	175.0612	175.0601	−6.49	C_7_H_12_O_5_	MS^2^[175]: 115.0386(100), 85.0644(42), 113.0594(38)		2‐Isopropylmalic acid isomer	Organic acid
50.	4.28	503.1406	503.1403	−0.61	C_21_H_28_O_14_	MS^2^[503]: 161.0232(100), 179.0339(42), 341.0874(37), 135.0438(31)		Caffeic acid‐O‐lactoside	Phenylpropanoid
51.	4.31	299.0772	299.0768	−1.34	C_13_H_16_O_8_	MS^2^[299]: 137.0231(100), 93.0332(77)		4‐Hydroxybenzoic acid β‐glucoside	Organic acid
52.	4.40	341.0878	341.0874	−1.10	C_15_H_18_O_9_	MS^2^[341]: 161.0232(100), 135.0438(41), 179.0338(40), 59.0124(17)		Caffeic acid‐O‐glucoside	Phenylpropanoid
53.	4.44	371.0984	371.0979	−1.19	C_16_H_20_O_10_	MS^2^[371]: 119.0489(100), 163.0389(78)		P‐Coumaric acid derivative	Phenylpropanoid
54.	4.56	299.0772	299.0770	−0.91	C_13_H_16_O_8_	MS^2^[299]: 137.0230(100), 93.0331(80)		4‐Hydroxybenzoic acid β‐glucoside	Organic acid
55.	4.58	339.0722	339.0718	−1.02	C_15_H_16_O_9_	MS^2^[339]: 177.0182(100), 133.0283(8), 339.0707(7)		Esculin isomer	Phenylpropanoid
56.	4.63	503.1406	503.1403	−0.61	C_21_H_28_O_14_	MS^2^[503]: 179.0339(100), 135.0438(82), 281.0663(67), 161.0232(60), 221.0447(48)		Caffeic acid‐O‐lactoside	Phenylpropanoid
57.	4.70	325.0929	325.0927	−0.74	C_15_H_18_O_8_	MS^2^[325]: 119.0488(100), 163.0388(39)		Coumaroylhexose	Phenylpropanoid
58.	4.70	207.0299	207.0290	−4.52	C_10_H_8_O_5_	MS^2^[207]: 163.0392(100), 135.0437(36), 118.9917(23)		MBQP isomer	Phenylpropanoid
59.	4.70	451.1246	451.1240	−1.39	C_21_H_24_O_11_	MS^2^[451]: 245.0815(100), 289.0716(97), 109.0282(63), 125.0231(56)		Catechin‐O‐glucoside	Flavonoid
60.	4.74	327.1085	327.1083	−0.89	C_15_H_20_O_8_	MS^2^[327]: 165.0545(100), 121.0644(9)		Dihydrocoumaroyl‐O‐glucoside	Phenylpropanoid
61.	4.78	341.0878	341.0874	−1.28	C_15_H_18_O_9_	MS^2^[341]: 135.0438(100), 179.0338(86)		Caffeic acid‐O‐glucoside	Phenylpropanoid
62.	4.81	503.1406	503.1403	−0.67	C_21_H_28_O_14_	MS^2^[503]: 179.0338(100), 135.0439(96), 161.0232(73), 221.0449(60)		Caffeic acid‐O‐lactoside	Phenylpropanoid
63.	4.81	339.0722	339.0717	−1.37	C_15_H_16_O_9_	MS^2^[339]: 177.0182(100), 133.0282(10), 339.0716(10)		Esculin isomer	Phenylpropanoid
64.	4.86	207.0299	207.02901	−3.99	C_10_H_8_O_5_	MS^2^[207]: 163.0389(100), 135.0437(31), 118.9914(20)		MBQP isomer	Phenylpropanoid
65.	4.91	435.0933	435.0930	−0.75	C_20_H_20_O_11_	MS^2^[435]: 137.0231(100), 289.0712(85), 109.0282(70)		Catechin‐O‐xyluronate	Flavonoid
66.	4.92	325.0929	325.0926	−1.02	C_15_H_18_O_8_	MS^2^[325]:119.0490(50), 93.0333(48)		Coumaroylhexose	Phenylpropanoid
67.	4.92	327.1085	327.1083	−0.89	C_15_H_20_O_8_	MS^2^[327]: 165.0545(100), 121.0646(10)		Dihydrocoumaroyl‐O‐glucoside	Phenylpropanoid
68.	4.99	272.1281	272.1287	2.10	C_16_H_17_NO_3_		MS^2^[272]: 107.0496(100), 161.05991(20), 143.0494(11), 237.0912(2)	Norcoclaurine isomer	Alkaloid
69.	5.04	563.1618	563.1613	−0.90	C_23_H_32_O_16_	MS^2^[563]:193.0496(100), 191.0703(40), 175.0390(40)		Ferulic acid‐O‐derivative	Phenylpropanoid
70.	5.05	341.0878	341.0874	−1.28	C_15_H_18_O_9_	MS^2^[341]: 135.0438(100), 179.0339(91), 161.0232(85), 281.0662(59), 89.0229(31)		Caffeic acid‐O‐glucoside	Phenylpropanoid
71.	5.05	517.1563	517.1556	−1.24	C_22_H_30_O_14_	MS^2^[517]: 161.0231(100), 355.1029(59), 193.0498(54), 134.0364(32), 133.0282(28)		Ferulic acid‐O‐lactoside	Phenylpropanoid
72.	5.10	272.1281	272.1289	2.68	C_16_H_17_NO_3_		MS^2^[272]: 107.0496(100), 143.0493(11), 161.0598(9), 237.0912(2)	Norcoclaurine isomer	Alkaloid
73.	5.11	665.1935	665.1934	−0.14	C_27_H_38_O_19_	MS^2^[665]: 161.0231(100), 503.1398(41), 179.0336(40), 135.0438(34)		Caffeic acid‐O‐gentianoside	Phenylpropanoid
74.	5.21	330.1700	330.1709	2.68	C_19_H_23_NO_4_		MS^2^[330]: 58.0658(100), 143.0490(12), 175.0755(10), 137.0599(8), 192.1019(3)	Reticulin isomer	Alkaloid
75.	5.24	503.1406	503.1404	−0.43	C_21_H_28_O_14_	MS^2^[503]: 179.0338(100), 221.0447(64), 161.0231(61)		Caffeic acid‐O‐lactoside	Phenylpropanoid
76.	5.30	289.0718	289.0715	−1.01	C_15_H_14_O_6_	MS^2^[289]: 109.0281(100), 123.0438(74), 125.0231(37), 97.0282(34)		Catechin isomer	Flavonoid
77.	5.32^a^	577.1351	577.1350	−0.24	C_30_H_26_O_12_	MS^2^[577]: 125.0232(100), 289.0717(37), 137.0232(25), 109.0283(20)		Procyanidin B2	Flavonoid
78.	5.41	503.1406	503.1403	−0.73	C_21_H_28_O_14_	MS^2^[503]: 179.0338(100), 135.0438(96), 161.0231(76), 221.0447(65)		Caffeic acid‐O‐lactoside	Phenylpropanoid
79.	5.49	325.0929	325.0926	−0.83	C_15_H_18_O_8_	MS^2^[325]: 119.0488(100), 163.0388(51)		Coumaroylhexose	Phenylpropanoid
80.	5.51^a^	289.0718	289.0715	−0.90	C_15_H_14_O_6_	MS^2^[289]: 109.0282(100), 123.0439(80), 97.0281(32), 125.0230(30)		Catechin	Flavonoid
81.	5.52	300.1594	300.1602	−0.99	C_18_H_21_NO_3_		MS^2^[300]: 58.0661(100), 107.0498(14), 223.0766(1), 175.0998(1)	N‐Methylcoclaurine isomer	Alkaloid
82.	5.53	517.1563	517.1558	−1.00	C_22_H_30_O_14_	MS^2^[517]: 295.0819(100), 193.0494(87), 175.0389(78), 134.0359(53)		Ferulic acid‐O‐lactoside	Phenylpropanoid
83.	5.54	448.1966	448.1977	2.45	C_23_H_29_NO_8_		MS^2^[448]: 107.0497(100), 269.1178(21), 175.0758(16), 143.0495(12), 286.1444(9)	Coclaurine‐O‐glucoside	Alkaloid
84.	5.59	341.0878	341.0873	−1.45	C_15_H_18_O_9_	MS^2^[341]: 135.0438(100), 179.0338(83)		Caffeic acid‐O‐glucoside	Phenylpropanoid
85.	5.59	325.0929	325.0925	−1.20	C_15_H_18_O_8_	MS^2^[325]: 119.0488(100), 163.0388(55)		Coumaroylhexose	Phenylpropanoid
86.	5.67	341.0878	341.0873	−1.63	C_15_H_18_O_9_	MS^2^[341]: 135.0438(100), 59.0124(60), 71.0124(37), 89.02309(27), 179.0339(22)		Caffeic acid‐O‐glucoside	Phenylpropanoid
87.	5.70	355.1035	355.1030	−1.28	C_16_H_20_O_9_	MS^2^[355]: 134.0360(100), 193.0496(53), 149.0595(24), 178.0261(16)		Ferulic acids‐O‐glucoside	Phenylpropanoid
88.	5.75	517.1563	517.1558	−0.89	C_22_H_30_O_14_	MS^2^[517]: 193.0498(100), 175.0390(72), 160.0157(56), 134.0359(54)		Ferulic acid‐O‐lactoside	Phenylpropanoid
89.	5.76	300.1594	300.1602	−0.99	C_18_H_21_NO_3_		MS^2^[300]: 107.0498(100), 58.0661(91), 237.0910(2)	N‐Methylcoclaurine isomer	Phenylpropanoid
90.	5.80	355.1035	355.1030	−1.37	C_16_H_20_O_9_	MS^2^[355]: 134.0360(100), 193.0496(62), 149.0595(27), 178.0261(17)		Ferulic acids‐O‐glucoside	Phenylpropanoid
91.	5.81	207.0299	207.0289	−4.67	C_10_H_8_O_5_	MS^2^[207]: 137.0231(100), 135.0434(39), 163.0387(35), 118.9916(24)		MBQP isomer	Phenylpropanoid
92.	5.84	177.0193	177.0182	−6.23	C_9_H_6_O_4_	MS^2^[177]: 133.0281(100), 105.0331(53), 89.0382(38), 149.0231(17)		Esculetin	Phenylpropanoid
93.	5.84	314.1387	314.1396	2.88	C_18_H_19_NO_4_		MS^2^[314]: 58.0661(100), 178.0867(86), 107.0498(34), 269.1178(5), 237.0915(4)	Didemethylation of magnoflorine	Alkaloid
94.	5.87^a^	330.1700	330.1708	2.59	C_19_H_23_NO_4_		MS^2^[330]: 58.0660(100), 239.0704(51), 181.0649(46), 223.0755(43), 213.0911(24)	Sinomenine	Alkaloid
95.	5.95	593.2087	593.2083	−0.05	C_25_H_38_O_16_	MS^2^[593]: 431.1554(100), 161.0442(26), 269.1035(17)		Zizybeoside II isomer	Other components
96.	5.96	445.1352	445.1348	−0.79	C_19_H_26_O_12_	MS^2^[445]: 137.0231(100), 93.0331(78)		4‐Hydroxybenzoic acid‐O‐rutinoside	Organic acid
97.	6.01	517.1563	517.1560	−0.52	C_22_H_30_O_14_	MS^2^[517]: 134.0362(100), 193.0495(89), 160.0155(66), 175.0390(65)		Ferulic acid‐O‐lactoside	Phenylpropanoid
98.	6.02	593.1723	593.1721	−0.44	C_24_H_34_O_17_	MS^2^[593]: 265.0713(100), 325.0925(78), 223.0604(47), 205.0499(39)		Sinapinic acid derivative	Phenylpropanoid
99.	6.05	313.0929	313.0928	−0.39	C_14_H_18_O_8_	MS^2^[313]: 167.0338(100), 108.0203(68), 123.0438(47), 152.0103(41)		Vanillic acid‐O‐rhamnoside	Organic acid
100.	6.06	451.1246	451.1243	−0.57	C_21_H_24_O_11_	MS^2^[451]: 289.0715(100), 245.0816(91), 125.0231(46)		Catechin‐O‐glucoside	Flavonoid
101.	6.10^a^	179.0350	179.0340	−5.49	C_9_H_8_O_4_	MS^2^[179]: 135.0438(100), 179.0338(17)		Caffeic acid	Phenylpropanoid
102.	6.13	302.1387	302.1396	0.91	C_17_H_19_NO_4_		MS^2^[302]: 123.0446(100), 143.0496(42), 175.0759(19), 285.1130(12)	Hydroxylation of coclaurine	Alkaloid
103.	6.19	577.1351	577.1349	−0.45	C_30_H_26_O_12_	MS^2^[577]: 125.0232(100), 289.0716(39), 161.0234(30), 109.0281(15)		Procyanidin B2 isomer	Flavonoid
104.	6.21	316.1543	316.1553	2.96	C_18_H_21_NO_4_		MS^2^[316]:58.0660(100), 192.1021(3),175.0755(3), 143.0493(3)	Norreticuline isomer	Alkaloid
105.	6.23	325.0930	325.0925	−1.20	C_15_H_18_O_8_	MS^2^[325]: 145.0282(100), 119.0490(80), 163.0388(72)		Coumaroylhexose	Phenylpropanoid
106.	6.23	593.2087	593.2079	−1.38	C_25_H_38_O_16_	MS^2^[593]: 431.1552(100), 269.1027(24), 161.0442(32)		Zizybeoside II isomer	Other components
107.	6.26	356.1498	356.1502	−0.30	C_20_H_22_O_5_N^+^		MS^2^[356]: 58.0661(100), 311.0921(2), 285.1128(1)	(S)‐4‐Keto‐magnoflorine isomer	Alkaloid
108.	6.30	475.1457	475.1453	−0.89	C_20_H_28_O_13_	MS^2^[475]: 167.0339(100), 152.0103(72), 108.0203(31), 123.0440(16)		Vanillic acid‐O‐rutinoside	Organic acid
109.	6.33	314.1751	314.1757	0.32	C_19_H_24_NO_3_^+^		MS^2^[314]: 58.0661(100), 107.0497(80), 269.1178(7), 237.0913(4)	Magnocurarine isomer	Alkaloid
110.	6.37	385.1657	385.1629	−5.67	C_22_H_26_O_6_	MS^2^[385]: 149.0231(100), 164.0467(94), 223.0605(94), 179.0705(28), 208.0367(28)		Sinapinic acid‐O‐glucoside	Phenylpropanoid
111.	6.41	151.0401	151.0388	−8.19	C_8_H_8_O_3_	MS^2^[151]: 136.0154(100), 123.0438(49), 108.0202(24)		Vanillin isomer	Organic acid
112.	6.45	356.1498	356.1502	−0.38	C_20_H_22_O_5_N^+^		MS^2^[356]: 58.0661(100), 311.0924(2), 285.1118(1)	(S)‐4‐Keto‐magnoflorine isomer	Alkaloid
113.	6.46	193.0506	193.0497	−4.93	C_10_H_10_O_4_	MS^2^[193]: 134.0360(100), 178.0260(35), 121.0278(19)		Ferulic acid isomer	Phenylpropanoid
114.	6.47	344.1862	344.1864	−0.88	C_20_H_26_NO_4_^+^		MS^2^[344]: 58.0661(100), 137.0601(33), 143.0496(19), 175.0759(11), 299.1288(4)	(S)‐Tembetarine isomer	Alkaloid
115.	6.49	595.1663	595.1666	−0.48	C_27_H_32_O_15_	MS^2^[595]: 355.0819(100), 385.0924(68), 475.1240(27), 415.1027(19)		Naringenin‐6,8‐di‐C‐glucoside	Flavonoid
116.	6.51	314.1387	314.1396	2.79	C_18_H_19_NO_4_		MS^2^[314]: 58.0661(100), 107.0498(37), 178.0868(10), 269.1178(5), 237.0918(3)	Didemethylation of magnoflorine	Alkaloid
117.	6.52	355.1035	355.1029	−1.54	C_16_H_20_O_9_	MS^2^[355]: 175.0389(100), 193.0496(17), 134.0360(12), 295.0818(2)		Ferulic acid‐O‐glucoside	Phenylpropanoid
118.	6.52	343.1035	343.1030	−1.24	C_15_H_20_O_9_	MS^2^[343]: 197.0444(100) 138.0310(52), 182.0210(46), 123.0074(41), 153.0547(32)		Syringic acid‐O‐rhamnoside	Alkaloid
119.	6.54	328.1543	328.1552	2.49	C_19_H_21_NO_4_		MS^2^[328]: 265.0865(100), 58.0661(79), 237.0916(13), 283.0973(12)	Desmethyl magnoflorine	Alkaloid
120.	6.57	344.1492	344.1504	1.14	C_19_H_21_NO_5_		MS^2^[344]: 58.0661(100), 137.0601(33), 143.0495(17), 175.0758(11), 299.1282(3)	Caulophine isomer	Alkaloid
121.	6.58	457.0988	457.0986	−0.29	C_19_H_22_O_13_	MS^2^[457]: 179.0338(100), 135.0438(79), 341.0870(11)		Caffeic acid‐O‐arabinoside‐ rhamnoside	Phenylpropanoid
122.	6.58	272.1281	272.1289	3.01	C_16_H_17_NO_3_		MS^2^[272]: 107.0496(100), 143.0494(13), 161.0599(8), 237.0913(2)	Norcoclaurine isomer	Alkaloid
123.	6.61	771.1989	771.1989	−0.03	C_33_H_40_O_21_	MS^2^[771]:300.02686(100), 271.0243(72), 151.0024(16), 301.0347(10)		Quercetin‐O‐glucoside‐rutinoside	Flavonoid
124.	6.62	316.1543	316.1552	2.77	C_18_H_21_NO_4_		MS^2^[316]: 107.0496(100), 58.0660(24), 192.1021(13), 175.0756(5), 143.492(4), 137.0602(2)	Norreticuline isomer	Alkaloid
125.	6.65	625.1410	625.1415	0.83	C_27_H_30_O_17_	MS^2^[625]: 299.0197(100), 301.0353(57), 271.0247(45), 300.0268(15)		Quercetin‐O‐lactoside	Flavonoid
126.	6.66	887.2463	887.2462	−0.14	C_38_H_48_O_24_	MS^2^[887]: 284.0325(100), 255.0295(89), 227.0339(34), 725.1946(28)		Kaempferol‐O‐rutinoside‐arapaioside‐glucoside	Flavonoid
127.	6.69	325.0929	325.0926	−0.83	C_15_H_18_O_8_	MS^2^[325]: 145.0282(100), 163.0388(80)		Coumaroylhexose	Phenylpropanoid
128.	6.70	358.1654	358.1658	−0.55	C_20_H_24_O_5_N		MS^2^[358]: 58.0661(100), 313.1078(27), 281.0808(22), 253.0871(17)	N‐Methyllaudanidinium isomer	Alkaloid
129.	6.71	303.0510	303.0508	−0.71	C_15_H_12_O_7_	MS^2^[303]: 125.0232(100), 177.0183(24), 217.0500(6), 285.0405(2)		Taxifolin isomer	Flavonoid
130.	6.72	771.1989	771.1987	−0.26	C_33_H_40_O_21_	MS^2^[771]: 300.0276(100), 271.0250(68), 301.0348(29), 151.0027(13)		Quercetin‐O‐glucoside‐rutinoside	Flavonoid
131.	6.73	356.1498	356.1502	−0.38	C_20_H_22_O_5_N^+^		MS^2^[356]: 58.0661(100), 311.0924(2), 285.1124(1)	(S)‐4‐Keto‐magnoflorine isomer	Alkaloid
132.	6.79	449.1089	449.1085	−0.92	C_21_H_22_O_11_	MS^2^[449]:259.0607(100), 269.0451(78), 287.0554(50)		Maesopsin 4‐O‐glucoside	Flavonoid
133.	6.79	328.1543	328.1550	1.94	C_19_H_21_NO_4_		MS^2^[328]: 58.0661(100), 265.0865(45), 283.0962(15), 237.0918(10)	Desmethyl magnoflorine	Alkaloid
134.	6.81	289.0718	289.0715	−0.90	C_15_H_14_O_6_	MS^2^[289]:123.0438(100), 109.0282(92), 125.0230(38), 97.0281(35)		Epicatechin	Flavonoid
135.	6.82	435.1297	435.1290	−1.47	C_21_H_24_O_10_	MS^2^[435]: 273.0765(100), 123.0441(9), 137.0234(7)		Phlorizin isomer	Flavonoid
136.	6.82	286.1438	286.1444	0.11	C_17_H_19_NO_3_		MS^2^[286]: 107.0498(100), 269.1178(14), 175.0758(10), 143.0495(10)	Coclaurine	Alkaloid
137.	6.86	328.1543	328.1551	2.21	C_19_H_21_NO_4_		MS^2^[328]: 58.0661(100), 265.0865(23), 283.0969(11), 237.0910(3)	Desmethyl magnoflorine	Alkaloid
138.	6.87	917.2568	917.2563	−0.60	C_39_H_50_O_25_	MS^2^[917]: 285.0401(100), 284.0351(42), 255.0278(30)		Kaempferol‐O‐dirutinoside	Flavonoid
139.	6.90	355.1035	355.1032	−0.69	C_16_H_20_O_9_	MS^2^[355]: 134.0360(100) 193.0496(87), 175.0389(86), 295.0819(59), 149.0596(27)		Ferulic acids‐O‐glucoside	Phenylpropanoid
140.	6.91	358.1654	358.1657	−0.80	C_20_H_24_O_5_N		MS^2^[358]: 58.0661(100), 313.1075(14), 281.0814(12), 253.0863(9)	N‐Methyllaudanidinium isomer	Alkaloid
141.	6.92	281.1391	281.1391	−1.13	C_15_H_22_O_5_	MS^2^[281]: 171.1170(100), 189.1279(23)		Dihydrophaseic acid	Other components
142.	6.93	449.1103	449.1092	−2.41	C_22_H_18_N_4_O_7_	MS^2^[449]: 125.0233(100), 269.0454(80), 259.0608(65), 287.0565(28)		N,N′‐[(2‐methoxyphenyl)methylene]bis(4‐nitrobenzamide)	Other components
143.	6.95	755.2040	755.2040	0.03	C_33_H_40_O_20_	MS^2^[755]: 284.0324(100), 255.0294(97), 285.0400(76), 593.1503(42), 227.0346(34)		Kaempferol‐O‐rutinoside‐glucoside	Flavonoid
144.	6.95	314.1387	314.1393	1.90	C_18_H_19_NO_4_		MS^2^[314]: 297.1127(100), 58.0661(85), 265.0864(78), 237.0914(30)	Didemethylation of magnoflorine	Alkaloid
145.	6.97	328.1543	328.1551	2.30	C_19_H_21_NO_4_		MS^2^[328]: 58.0661(100), 265.0863(22), 283.0970(14), 237.0909(3)	Desmethyl magnoflorine	Alkaloid
146.	6.99	445.1352	445.1354	0.52	C_19_H_26_O_12_	MS^2^[445]: 121.0283(100), 135.0440(15)		1’‐O‐benzoylsucrose	Alkaloid
147.	7.03	327.1085	327.1082	−1.10	C_15_H_20_O_8_	MS^2^[327]: 165.0545(100)		Dihydrocoumaroyl‐O‐glucoside	Phenylpropanoid
148.	7.07	448.1966	448.1979	2.87	C_23_H_29_NO_8_		MS^2^[448]: 107.0498(100), 269.1179(19), 143.0496(17) 286.1444(13), 175.0759(12)	Coclaurine‐O‐glucoside	Alkaloid
149.	7.08	165.0552	165.0546	−6.95	C_9_H_10_O_3_	MS^2^[165]: 147.0439(100), 72.9916(35), 119.0488(32)		3‐Phenyllactic acid	Organic acid
150.	7.10	434.1809	434.1822	2.94	C_22_H_27_NO_8_		MS^2^[434]: 107.0498(100), 255.1021(24), 272.1286(22), 143.0495(20)	Norcoclaurine‐O‐glucoside	Alkaloid
151.	7.15	179.0350	179.0339	−5.82	C_9_H_8_O_4_	MS^2^[179]: 109.0281(100), 135.0439(44), 59.0124(35), 71.0123(27)		Caffeic acid isomer	Phenylpropanoid
152.	7.15	358.1654	358.1659	−0.21	C_20_H_24_O_5_N		MS^2^[358]: 58.0661(100), 295.0973(64), 235.0760(55), 313.1076(47), 340.1543(22), 281.0825(3)	N‐Methyllaudanidinium isomer	Alkaloid
153.	7.16	302.1387	302.1396	0.91	C_17_H_19_NO_4_		MS^2^[302]: 107.0498(100), 285.1129(11)	Hydroxylation of coclaurine	Alkaloid
154.	7.19	625.1410	625.1431	3.28	C_27_H_30_O_17_	MS^2^[625]: 316.0223(100), 271.0247(80), 299.0194(55), 125.0231(29), 317.0302(29),		Myricetin‐O‐rutinoside	Flavonoid
155.	7.20	316.1543	316.1553	2.96	C_18_H_21_NO_4_		MS^2^[316]: 137.0599(100), 143.04949(70), 175.0756(25)	Norreticuline isomer	Alkaloid
156.	7.21	330.1670	330.1709	2.68	C_19_H_23_NO_4_		MS^2^[330]: 58.0660(100), 143.0494(37), 137.0599(30), 175.0756(25), 192.1019(11)	Reticuline isomer	Alkaloid
157.	7.22	755.2040	755.2040	0.03	C_33_H_40_O_20_	MS^2^[755]: 285.0403(100), 255.0296(51), 284.0322(38), 593.1516(29), 227.0348(18)		Kaempferol‐O‐rutinoside‐glucoside	Flavonoid
158.	7.22	344.1492	344.1507	1.42	C_19_H_21_NO_5_		MS^2^[344]: 58.0661(100), 137.0601(33), 143.0495(18), 175.0758(11), 299.1282(4)	Caulophine isomer	Alkaloid
159.	7.24	167.0350	167.0339	−6.72	C_8_H_8_O_4_	MS^2^[167]: 152.0103(100), 124.0152(34), 108.0203(29)		Vanillic acid	Organic acid
160.	7.26	328.1543	328.1552	2.58	C_19_H_21_NO_4_		MS^2^[328]: 58.0661(100), 265.0869(21), 283.0967(11), 237.0921(3)	Desmethyl magnoflorine	Alkaloid
161.	7.27	355.1035	355.1031	−1.023	C_16_H_20_O_9_	MS^2^[355]: 134.0360(100), 193.0496(82), 295.0819(66), 149.0595(23)		Ferulic acids‐O‐glucoside	Phenylpropanoid
162.	7.29	344.1862	344.1864	−0.88	C_20_H_26_NO_4_^+^		MS^2^[344]: 58.0661(100), 137.0602(33), 143.0496(18), 175.0759(13), 299.1287(4)	(S)‐Tembetarine isomer	Alkaloid
163.	7.31	625.1410	625.1405	−0.84	C_27_H_30_O_17_	MS^2^[625]: 316.0223(100), 271.0247(53), 317.0298(33),		Myricetin‐O‐rutinoside	Flavonoid
164.	7.33	358.2013	358.2023	2.75	C_21_H_27_NO_4_		MS^2^[358]: 58.0660(100), 253.0862(7), 313.1073(6)	Methylation of magnoflorine	Alkaloid
165.	7.36	282.1125	282.1133	2.87	C_17_H_15_NO_3_		MS^2^[282]: 156.0448(100), 188.0712(88), 128.0499(61)	Juzirine	Alkaloid
166.	7.43	328.1543	328.1552	2.49	C_19_H_21_NO_4_		MS^2^[328]: 58.0661(100), 265.0865(27), 283.0985(12), 237.0919(6)	Desmethyl magnoflorine	Alkaloid
167.	7.43	344.1492	344.1506	1.32	C_19_H_21_NO_5_		MS^2^[344]: 58.0661(100), 137.0602(34), 143.0496(18), 175.0760(11), 299.1296(4)	Caulophine isomer	Alkaloid
168.	7.43	312.1594	312.1603	2.66	C_19_H_22_NO_3_		MS^2^[312]: 58.0660(100), 267.1017(14), 235.0755(11), 207.0807(6)	Demethoxylation of magnoflorine	Alkaloid
169.	7.46	400.1755	400.1765	2.61	C_22_H_25_NO_6_		MS^2^[400]:58.0660(100), 323.0916(30), 355.1179(18), 295.0967(5)	C10‐OCH3‐ Hydroxylation of M16	Alkaloid
170.	7.48	358.1654	358.1656	−1.05	C_20_H_24_O_5_N		MS^2^[358]: 58.0661(100), 313.1078(36), 281.0815(9), 253.0866(6)	N‐Methyllaudanidinium isomer	Alkaloid
171.	7.49	625.1410	625.1411	0.04	C_27_H_30_O_17_	MS^2^[625]: 316.0.222(100), 317.0294(41), 271.0247(34), 287.0210(17)		Myricetin‐O‐rutinoside	Flavonoid
172.	7.57^a^	342.1705	342.1707	2.03	C_20_H_24_NO_4_^+^		MS^2^[342]: 58.0661(100), 265.0866(28), 297.1128(25), 282.0894(13), 237.0916(9), 191.0849(9)	Magnoflorine	Alkaloid
173.	7.64	625.1410	625.1401	−1.51	C_27_H_30_O_17_	MS^2^[625]: 316.0223(100), 317.0298(44), 109.9996(20), 271.0247(9), 125.0233(9)		Myricetin‐O‐rutinoside	Flavonoid
174.	7.76	151.0401	151.0389	−7.53	C_8_H_8_O_3_	MS^2^[151]: 136.0152(100), 123.0441(65), 108.0202(49)		Vanillin isomer	Organic acid
175.	7.77	179.0350	179.0341	−5.15	C_9_H_8_O_4_	MS^2^[179]: 135.0439(100), 59.0124(53), 71.0124(36)		Caffeic acid isomer	Phenylpropanoid
176.	7.77	330.1700	330.1714	4.17	C_19_H_23_NO_4_		MS^2^[330]: 192.1021(100), 137.0599(58), 143.0494(30), 58.0660(21), 175.0756(20)	Reticuline isomer	Alkaloid
177.	7.79	771.1989	771.1992	0.30	C_33_H_40_O_21_	MS^2^[771]: 300.0268(100), 301.0348(62), 271.0244(38), 255.0297(32), 151.0026(28)		Quercetin‐O‐glucoside‐rutinoside	Flavonoid
178.	7.94	771.1989	771.1992	0.30	C_33_H_40_O_21_	MS^2^[771]: 300.0262(100), 301.0349(68), 151.0028(51), 271.0247(28), 255.0290(23)		Quercetin‐O‐glucoside‐rutinoside	Flavonoid
179.	7.98	314.1751	314.1758	0.61	C_19_H_24_NO_3_^+^		MS^2^[314]: 107.0497(100), 58.0661(58), 143.0496(16), 269.1176(12), 237.0917(7)	Magnocurarine isomer	Alkaloid
180.	8.02	433.1140	433.1135	−1.13	C_21_H_22_O_10_	MS^2^[433]: 313.0716(100), 271.0611(33), 343.0819(26)		Naringenin‐C‐glucoside	Flavonoid
181.	8.13	625.1410	625.1414	0.54	C_27_H_30_O_17_	MS^2^[625]: 316.0224(100), 317.0299(23), 271.0247(22)		Myricetin‐O‐rutinoside	Flavonoid
182.	8.13	358.2013	358.2023	2.92	C_21_H_27_NO_4_		MS^2^[358]: 58.0660(100), 253.0863(3), 281.0815(2)	Methylation of magnoflorine	Alkaloid
183.	8.15	755.2040	755.2038	−0.29	C_33_H_40_O_20_	MS^2^[755]: 300.0275(100), 271.0247(34), 255.0297(14), 301.0324(10)		Quercetin‐O‐rutinoside‐rhamnoside	Flavonoid
184.	8.22	457.1351	457.1348	−0.70	C_20_H_26_O_12_	MS^2^[457]: 163.0388(100), 119.0488(95)		P‐Coumaric acid‐O‐glucoside‐arapaioside	Phenylpropanoid
185.	8.22	741.1884	741.1882	−0.18	C_32_H_38_O_20_	MS^2^[741]: 300.0279(100), 271.0246(53), 255.0290(31), 243.290(11), 301.0325(5)		Quercetin‐O‐rutinoside‐arapaioside	Flavonoid
186.	8.22	340.1543	340.1553	2.93	C_20_H_21_NO_4_		MS^2^[340]: 263.0709(100), 295.0973(88), 235.0758(54), 58.0661(35)	C4,C5‐Dehydrogenation of magnoflorine isomer	Alkaloid
187.	8.29	433.1140	433.1136	−0.92	C_21_H_22_O_10_	MS^2^[433]: 313.0717(100), 119.0487(63), 125.0232(26), 343.0826(26), 271.0608(17)		Naringenin‐C‐glucoside	Alkaloid
188.	8.29	300.1594	300.1603	−0.79	C_18_H_21_NO_3_		MS^2^[300]: 107.0498(100), 237.0913(8)	N‐Methylcoclaurine isomer	Alkaloid
189.	8.33	358.2013	358.2021	2.16	C_21_H_27_NO_4_		MS^2^[358]: 58.0660(100), 281.0814(2)	Methylation of magnoflorine	Alkaloid
190.	8.35	771.1989	771.1986	−0.49	C_33_H_40_O_21_	MS^2^[771]: 300.0275(100), 301.0351(90), 271.0248(46), 151.0025(37)		Quercetin‐O‐glucoside‐rutinoside	Flavonoid
191.	8.43	312.1594	312.1605	3.33	C_19_H_22_NO_3_		MS^2^[312]: 267.1018(100), 58.0660(36), 107.0497(23)	Demethoxylation of magnoflorine	Alkaloid
192.	8.47^a^	193.0506	193.0497	−4.93	C_10_H_10_O_4_	MS^2^[193]: 134.0361(100), 178.0261(61), 193.0496(48), 149.0595(28)		Ferulic acid	Phenylpropanoid
193.	8.47	433.1140	433.1140	−0.09	C_21_H_22_O_10_	MS^2^[433]: 313.0714(100), 151.0025(46), 271.0610(40), 343.0827(22)		Naringenin‐C‐glucoside	Flavonoid
194.	8.48	204.0666	204.0661	−2.73	C_11_H_11_O_3_N	MS^2^[204]: 158.0602(100), 116.0494(44), 142.0652(38), 186.0553(38), 130.0651(33)		Indole‐3‐lactic acid	Organic acid
195.	8.55	625.1410	625.1414	−0.44	C_27_H_30_O_17_	MS^2^[625]: 316.0222(100), 317.0293(22), 271.0247(21)		Myricetin‐O‐rutinoside	Flavonoid
196.	8.59	595.1305	595.1304	−0.15	C_26_H_28_O_16_	MS^2^[595]: 300.02750(100), 271.0236(53), 255.0291(21), 243.0303(15)		Quercetin‐O‐glucoside‐arabinoside	Flavonoid
197.	8.65	303.0510	303.0508	−0.71	C_15_H_12_O_7_	MS^2^[303]: 125.0232(100), 285.0410(50), 151.0024(15), 177.0183(15)		Taxifolin isomer	Flavonoid
198.	8.72	340.1543	340.1554	3.01	C_20_H_21_NO_4_		MS^2^[340]: 58.0661(35), 235.0763(10), 295.0977(8), 263.0703(5)	C4,C5‐Dehydrogenation of magnoflorine	Alkaloid
199.	8.75	319.0818	319.0820	−1.12	C_16_H_16_O_7_	MS^2^[319]: 119.0489(100), 163.0389(69), 93.0332(30), 155.0338(11), 111.0437(6)		Coumaroylshikimate	Phenylpropanoid
200.	8.80	281.1391	281.1392	−0.91	C_15_H_22_O_5_	MS^2^[281]: 171.1168(100), 237.1491(43), 189.1277(35)		Dihydrophaseic acid	Other components
201.	8.80	300.1594	300.1603	−0.69	C_18_H_21_NO_3_		MS^2^[300]: 107.0498(100), 143.0495(14), 269.1178(11), 237.0913(8), 192.1025(6)	N‐Methylcoclaurine isomer	alkaloid
202.	8.87	433.1140	433.1136	−0.99	C_21_H_22_O_10_	MS^2^[433]: 271.0609(100), 151.0025(80), 125.0231(25)		Naringenin‐O‐glucoside	Flavonoid
203.	8.89	417.1191	417.1187	−0.88	C_21_H_22_O_9_	MS^2^[417]: 255.0661(100), 119.0490(97), 135.0076(59), 153.0183(45)		Isoliquiritin isomer	Flavonoid
204.	8.92	411.1297	411.1295	−0.44	C_19_H_24_O_10_	MS^2^[411]: 119.0489(100), 163.0389(62)		P‐Coumaric acid derivative	Phenylpropanoid
205.	8.97	137.0244	137.0231	−9.47	C_7_H_6_O_3_	MS^2^[137]: 93.0331(100)		4‐Hydroxybenzoic acid	Organic acid
206.	8.98	268.1332	268.1339	2.74	C_17_H_17_NO_2_		MS^2^[268]: 191.0860(100), 219.0811(47), 251.1073(42), 236.0838(13)	(R)‐Asimilobine isomer	Alkaloid
207.	9.08	356.1856	356.1866	2.57	C_21_H_25_NO_4_		MS^2^[356]: 58.0660(100), 279.1019(37), 296.1046(11), 251.1068(9)	Methyl‐magnoflorine	Alkaloid
208.	9.11	609.1461	609.1457	−0.69	C_27_H_30_O_16_	MS^2^[609]: 300.0274(100), 271.0247(64), 255.0298(33), 301.0346(22)		Quercetin 3‐O‐rutinoside isomer	Flavonoid
209.	9.13	607.1305	607.1293	−1.86	C_27_H_28_O_16_	MS^2^[607]: 299.0193(100), 271.0244(66), 300.0269(33), 151.0026(16), 301.0360(15)		Quercetin‐O‐glucuronide‐rhamnoside	Flavonoid
210.	9.22	725.1935	725.1932	−0.38	C_32_H_38_O_19_	MS^2^[725]: 284.0324(100), 255.0293(74), 227.0343(40), 285.0398(19)		Kaempferol‐O‐rutinoside‐arapaioside	Flavonoid
211.	9.23	463.0882	463.0878	−0.86	C_21_H_20_O_12_	MS^2^[463]: 300.0276(100), 151.0025(31), 301.0352(26)		Isoquercitrin isomer	Flavonoid
212.	9.27^a^	609.1461	609.1458	−0.59	C_27_H_30_O_16_	MS^2^[609]: 300.0276(100), 271.0250(59), 255.0299(29), 301.0355(20)		Quercetin 3‐O‐rutinoside	Flavonoid
213.	9.29	607.1305	607.1292	−2.06	C_27_H_28_O_16_	MS^2^[607]: 299.0196(100), 271.0245(80), 300.0264(25), 151.0027(24), 243.0296(22)		Quercetin‐O‐glucuronide‐rhamnoside	Flavonoid
214.	9.35	755.2040	755.2035	−0.70	C_33_H_40_O_20_	MS^2^[755]: 314.0132(100), 299.0196(52), 227.0343(33), 315.0514(28), 285.0404(25), 271.0248(19)		Methylquercetin‐O‐rutinoside‐arapaioside	Flavonoid
215.	9.38^a^	463.0882	463.0880	−0.54	C_21_H_20_O_12_	MS^2^[463]: 300.0273(100), 301.0352(38), 271.0246(14), 255.0296(10)		Hyperoside	Flavonoid
216.	9.44	597.1825	597.1834	1.43	C_27_H_34_O_15_	MS^2^[597]: 357.0985(100), 387.1078(82), 167.0340(48), 209.0447(32), 315.0875(22)		3′, 5’‐Di‐C‐β‐D‐glucosylphloretin	Flavonoid
217.	9.45	137.0244	137.0231	−9.47	C_7_H_6_O_3_	MS^2^[137]: 93.0331(100)		4‐Hydroxybenzoic acid isomer	Organic acid
218.	9.52	151.0401	151.0389	−7.86	C_8_H_8_O_3_	MS^2^[151]: 136.0152(100), 108.0202(21), 123.0438(10)		Vanillin isomer	Organic acid
219.	9.57^a^	463.0882	463.0880	−0.34	C_21_H_20_O_12_	MS^2^[463]: 300.0268(100), 271.0244(56), 301.0346(44), 255.0295(29)		Isoquercitrin	Flavonoid
220.	9.69^a^	447.0933	447.0928	−1.02	C_21_H_20_O_11_	MS^2^[447]: 285.0403(100), 284.0321(41)		Luteolin 7‐O‐glucoside	Flavonoid
221.	9.75	679.1880	679.1876	−0.52	C_31_H_36_O_17_	MS^2^[679]: 161.0231(100), 193.0500(40), 517.1342(38), 135.0438(39), 179.0341(22)		Caffeoyl ferulic acid‐O‐lactoside	Phenylpropanoid
222.	9.81	663.1931	663.1929	−0.19	C_31_H_36_O_16_	MS^2^[663]: 119.0489(100), 501.1403(34), 163.0391(33), 175.0394(27)		P‐Coumaric acid‐O‐lactoside‐ glucuronide	Phenylpropanoid
223.	9.92^a^	593.1512	593.1510	−0.33	C_27_H_30_O_15_	MS^2^[593]: 284.0321(100), 255.0291(95), 227.0343(49), 285.0402(40)		Kaempferol‐3‐O‐neohesperidoside	Flavonoid
224.	9.94	435.1297	435.1295	−0.41	C_21_H_24_O_10_	MS^2^[435]: 273.0774(11), 167.0339(59), 125.0231(39), 123.0438(27), 179.0340(12)		Phlorizin isomer	Flavonoid
225.	10.19	268.1332	268.1339	2.74	C_17_H_17_NO_2_		MS^2^[268]: 251.1072(100), 219.0810(86), 191.0860(77)	(R)‐Asimilobine isomer	Alkaloid
226.	10.32	296.1645	296.1652	2.41	C_19_H_21_NO_2_		MS^2^[296]: 219.0807(100), 58.0660(84), 251.1069(84), 191.0857(49)	Nuciferine	Alkaloid
227.	10.34	447.0933	447.0929	−0.79	C_21_H_20_O_11_	MS^2^[447]: 284.0325(100), 255.0294(61), 227.0345(24), 285.0407(19)		Kaempferol‐O‐glucoside	Flavonoid
228.	10.36	635.1981	635.1977	−0.71	C_30_H_36_O_15_	MS^2^[635]: 119.0489(100), 163.0390(46)		P‐Coumaric acid derivative	Phenylpropanoid
229.	10.39	433.1140	433.1135	−1.13	C_21_H_22_O_10_	MS^2^[433]: 300.0273(100), 271.0607(70), 151.0026(39), 301.0337(25), 255.0299(18)		Quercetin‐O‐arapaioside	Flavonoid
230.	10.43	374.1598	374.1610	1.14	C_20_H_23_NO_6_		MS^2^[374]: 58.0661(100), 255.0658(22), 329.1025(20), 314.0799(4)	Chiloenamine	Alkaloid
231.	10.50^a^	593.1512	593.1509	−0.53	C_27_H_30_O_15_	MS^2^[593]: 284.0322(100), 285.0322(99), 255.0292(84), 227.0341(39)		Nicotiflorin	Flavonoid
232.	10.51	607.1668	607.1663	−0.86	C_28_H_32_O_15_	MS^2^[607]: 161.0236(100), 179.0340(74)		Caffeic acid derivative	Phenylpropanoid
233.	10.62	623.1618	623.1613	−0.82	C_28_H_32_O_16_	MS^2^[623]: 299.0184(100), 314.0430(91), 243.0288(43), 315.0517(40)		Methylquercetin‐O‐rutinoside	Flavonoid
234.	10.71	579.2083	579.2081	−0.32	C_28_H_36_O_13_	MS^2^[579]:181.0495(100), 417.1551(99), 402.1310(8)		Syringaresinol O‐β‐D‐glucoside	Phenylpropanoid
235.	10.78	679.1880	679.1873	−1.05	C_31_H_36_O_17_	MS^2^[679]: 161.0234(100), 179.0340(64), 135.0437(49)		Caffeic acid‐O‐lactoside‐ glucuronide	Phenylpropanoid
236.	10.85	579.1355	579.1351	−0.82	C_26_H_28_O_15_	MS^2^[579]: 300.0272(100), 271.0244(53), 255.0295(27), 243.0290(11), 301.0348(3)		Quercetin‐O‐rhamnoside‐arabinoside	Flavonoid
237.	10.88^a^	447.0933	447.0929	−0.86	C_21_H_20_O_11_	MS^2^[447]:300.0278(100), 301.0352(77), 271.0251(55), 255.0295(40), 107.0128(39), 151.0029(37), 243.0307(13)		Quercitrin	Flavonoid
238.	10.88	623.1618	623.1613	−0.70	C_28_H_32_O_16_	MS^2^[623]: 315.0505(100), 299.0209(44), 271.0242(39)		Methylquercetin‐O‐rutinoside	Flavonoid
239.	10.90	326.1751	326.1760	0.89	C_20_H_23_NO_3_		MS^2^[326]: 249.0916(100), 221.0967(55), 281.1179(38), 58.0661(13)	Dehydroxylation of magnoflorine	Alkaloid
240.	10.93	579.1719	579.1707	−2.09	C_27_H_32_O_14_	MS^2^[579]: 300.0275(100), 271.0241(60), 255.0296(20)		Quercetin derivatives	Flavonoid
241.	10.96	607.1668	607.1664	−0.66	C_28_H_32_O_15_	MS^2^[607]: 179.0340(100), 221.0448(85), 281.0666(80)		Caffeic acid derivative	Phenylpropanoid
242.	11.10	607.1668	607.1669	0.04	C_28_H_32_O_15_	MS^2^[607]: 281.0666(100), 179.0340(96), 221.0449(48), 161.0232(17)		Caffeic acid derivative	Phenylpropanoid
243.	11.13	579.1355	579.1352	−0.61	C_26_H_28_O_15_	MS^2^[579]: 300.0272(100), 271.0244(56), 255.0295(28), 243.0293(11), 301.0311(8)		Quercetin‐O‐rhamnoside‐arabinoside	Flavonoid
244.	11.32	607.1668	607.1666	−0.47	C_28_H_32_O_15_	MS^2^[607]: 179.0339(100), 281.0667(92), 221.0449(74), 251.0558(26)		Caffeic acid derivative	Phenylpropanoid
245.	11.38^a^	435.1297	435.1292	−1.20	C_21_H_24_O_10_	MS^2^[435]: 167.0339(100), 123.04389(45), 273.0767(23), 125.02313(20), 179.0337(12)		Phlorizin	Flavonoid
246.	11.52	517.1351	517.1347	−0.87	C_25_H_26_O_12_	MS^2^[517]: 193.0500(100), 175.0241(66), 179.0343(39), 135.0360(10)		Caffeoyl ferulic acid‐O‐glucoside	Phenylpropanoid
247.	11.53	607.1668	607.1669	0.14	C_28_H_32_O_15_	MS^2^[607]: 179.0340(100), 281.0666(99), 221.0449(48), 251.0559(46)		Caffeic acid derivative	Phenylpropanoid
248.	11.63	663.1931	663.1930	−0.10	C_31_H_36_O_16_	MS^2^[663]: 163.0394(100), 119.0486(30), 193.0503(21), 501.1393(18)		P‐Coumaric acid‐O‐lactoside‐ glucuronide	Phenylpropanoid
249.	11.96	607.1668	607.1659	−1.57	C_28_H_32_O_15_	MS^2^[607]: 179.0340(100)		Caffeic acid derivative	Phenylpropanoid
250.	12.05	447.0933	447.0928	−1.15	C_21_H_20_O_11_	MS^2^[447]: 285.0401(100), 300.0263(8)		Luteolin 7‐O‐glucoside isomer	Flavonoid
251.	12.19	661.1774	661.1774	−0.06	C_31_H_34_O_16_	MS^2^[661]: 161.0232(100), 175.0392(57), 193.0504(34), 179.0340(31)		Dicaffeoyl ferulic acid‐O‐rhamnoside	Phenylpropanoid
252.	12.42	517.1351	517.1346	−0.99	C_25_H_26_O_12_	MS^2^[517]: 135.0437(100), 179.0337(76), 134.0361(11), 175.0392(9)		Caffeoyl ferulic acid‐O‐glucoside	Phenylpropanoid
253.	12.60	433.1140	433.1138	−0.51	C_21_H_22_O_10_	MS^2^[433]: 167.0340(100), 152.0104(65), 108.0203(61), 123.0436(27)		Vanillic acid‐derivative	Organic acid
254.	12.69	282.1489	282.1495	2.32	C_18_H_19_NO_2_		MS^2^[282]: 265.1229(100), 250.0994(62), 234.1044(47), 219.0809(18)	O‐nornuciferine	Alkaloid
255.	12.80	445.0776	445.0771	−1.16	C_21_H_18_O_11_	MS^2^[445]: 269.0452(100), 113.0231(12), 85.0279(9)		Baicalin	Flavonoid
256.	12.81	263.1283	263.1285	−1.42	C_15_H_20_O_4_	MS^2^[263]: 219.1380(100), 151.0754(77), 201.1282(38), 152.0832(32), 203.1071(29)		Abscisic acid	Organic acid
257.	12.83	517.1351	517.1347	−0.87	C_25_H_26_O_12_	MS^2^[517]: 179.0341(100), 135.0440(16), 175.0239(4), 193.0500(2)		Caffeoyl ferulic acid‐O‐glucoside	Phenylpropanoid
258.	12.89^a^	435.1297	435.1294	0.86	C_21_H_24_O_10_	MS^2^[435]:273.0774(100), 167.0344(46), 125.0238(4)		Trilobatin	Flavonoid
259	12.98	417.1191	417.1187	−0.88	C_21_H_22_O_9_	MS^2^[417]: 255.0605(100), 119.0489(51), 135.0074(50), 153.0179(38)		Isoliquiritin isomer	Flavonoid
260.	13.13	721.1985	721.1983	−0.33	C_33_H_38_O_18_	MS^2^[721]: 175.0393(100), 193.0498(67), 134.0361(65)		Ferulic acid‐O‐derivative	Phenylpropanoid
261.	13.34	445.1140	445.1138	−0.56	C_22_H_22_O_10_	MS^2^[445]: 135.0438(100), 179.0340(72)		Caffeic acid derivative	Phenylpropanoid
262.	13.38	501.1402	501.1400	−0.57	C_25_H_26_O_11_	MS^2^[501]: 119.0490(100), 163.0390, (69), 175.0393(31)		P‐Coumaric acid‐O‐glucoside‐glucuronide	Phenylpropanoid
263.	13.43	417.1191	417.1190	−0.35	C_21_H_22_O_9_	MS^2^[417]: 119.0489(100), 255.0660(63), 135.0075(35), 153.0182(15)		Isoliquiritin isomer	Flavonoid
264.	13.62	531.1508	531.1506	−0.43	C_26_H_28_O_12_	MS^2^[531]: 193.0500(100), 134.0359(62), 178.0262(45)		Ferulic acid‐O‐glucoside‐glucuronide	Phenylpropanoid
265.	13.77	445.1140	445.1137	−0.76	C_22_H_22_O_10_	MS^2^[445]: 135.0439(100), 179.0339(75)		Caffeic acid derivative	Phenylpropanoid
266.	13.86	207.0663	207.0654	−4.36	C_11_H_12_O_4_	MS^2^[207]: 135.0439(100), 179.0340(53), 161.0234(42)		Caffeic acid ethyl ester	Phenylpropanoid
267.	14.24	501.1402	501.1401	−0.25	C_25_H_26_O_11_	MS^2^[501]: 119.0488(100), 163.0390(34), 175.0395(26)		P‐Coumaric acid‐O‐glucoside‐glucuronide	Phenylpropanoid
268.	14.26^a^	301.0354	301.0350	−1.35	C_15_H_10_O_7_	MS^2^[301]: 151.0026(100), 301.0351(86), 107.125(43), 178.9976(29)		Quercetin	Flavonoid
269.	14.66	505.1351	505.1351	−0.10	C_24_H_26_O_12_	MS^2^[505]: 149.0596(100), 193.0497(92), 134.0361(77)		Ferulic acid derivative	Phenylpropanoid
270.	15.24	445.1140	445.1136	−0.97	C_22_H_22_O_10_	MS^2^[445]: 135.0439(100), 179.0340(66)		Caffeic acid derivative	Phenylpropanoid
271.	15.71	459.0933	459.0929	−0.84	C_22_H_20_O_11_	MS^2^[459]: 268.0373(100), 283.0610(49), 113.0230(30)		Wogonin‐O‐glucuronide	Flavonoid
272.	18.05	329.2334	329.2334	0.25	C_18_H_34_O_5_	MS^2^[329]: 171.1018(100), 201.1126(66), 127.1117(27)		(15Z)‐9,12,13‐trihydroxy‐15‐octadecenoic acid	Other components
273.	19.31	227.1289	227.1286	−1.15	C_12_H_20_O_4_	MS^2^[227]: 183.1383(100), 165.1277(18)		Butanedioic acid isomer	Other components
274.	20.06	329.2334	329.2330	−0.96	C_18_H_34_O_5_	MS^2^[329]: 201.1121(100), 127.1114(17), 171.1013(15)		(15Z)‐9,12,13‐trihydroxy‐15‐octadecenoic acid	Other components
275.	20.34	229.1445	229.1439	−2.72	C_12_H_22_O_4_	MS^2^[229]: 229.1436(100), 167.1431(44)		Dodecanedioic acid	Other components
276.	20.43	329.2334	329.2329	−1.33	C_18_H_34_O_5_	MS^2^[329]: 171.1015(100), 127.117(25), 201.1128(4)		(15Z)‐9,12,13‐trihydroxy‐15‐octadecenoic acid	Other components
277.	21.17	329.2334	329.2330	−1.06	C_18_H_34_O_5_	MS^2^[329]: 171.1014(100), 127.1119(18)		(15Z)‐9,12,13‐trihydroxy‐15‐octadecenoic acid	Other components
278.	22.51	329.2334	329.2329	−1.24	C_18_H_34_O_5_	MS^2^[329]: 171.1015(100), 201.1123(63), 127.1115(23)		(15Z)‐9,12,13‐trihydroxy‐15‐octadecenoic acid	Other components
279.	23.22	535.1974	535.1971	−0.40	C_30_H_32_O_9_	MS^2^[535]: 119.0488(100), 145.0284(54), 163.0390(50)		P‐Coumaric acid derivative	phenylpropanoid
280	23.33	535.1974	535.1968	−0.96	C_30_H_32_O_9_	MS^2^[535]: 119.0488(100), 163.0388(62)		P‐Coumaric acid derivative	phenylpropanoid
281.	23.40	329.2334	329.2330	−1.06	C_18_H_34_O_5_	MS^2^[329]: 171.1014(100), 201.1130(62)		(15Z)‐9,12,13‐trihydroxy‐15‐octadecenoic acid	Other components
282.	23.42	821.3965	821.3970	0.56	C_42_H_62_O_16_	MS^2^[821]: 113.0231(100), 193.0345(22), 351.0569(16)		Glycyrrhizic acid	Other components
283.	26.49	499.3065	499.3061	−0.79	C_30_H_44_O_6_	MS^2^[499]: 437.0340(100)		3‐Dehydroxy ceanothetric acid	Other components
284.	27.02^a^	593.1301	593.1297	−0.61	C_30_H_26_O_13_	MS^2^[593]: 121.0281(100), 209.0446(31)		Proanthocyanidins	flavonoid
285.	27.18	499.3065	499.3061	−0.79	C_30_H_44_O_6_	MS^2^[499]: 437.3059(100), 455.3163(42)		3‐Dehydroxy ceanothetric acid isomer	Other components
286.	27.80	499.3065	499.3062	−0.73	C_30_H_44_O_6_	MS^2^[499]: 455.3161(100)		3‐Dehydroxy ceanothetric acid isomer	Other components
287.	28.45	499.3065	499.3060	−1.03	C_30_H_44_O_6_	MS^2^[499]: 437.3049(100)		3‐Dehydroxy ceanothetric acid isomer	Other components
288.	29.84	485.3272	485.3275	0.54	C_30_H_46_O_5_	MS^2^[485]: 423.3298(100)		Ceanothic acid isomer	Other components
289.	30.52	485.3272	485.3266	−1.34	C_30_H_46_O_5_	MS^2^[485]: 423.3268(100)		Ceanothic acid isomer	Other components
290.	31.12	485.3272	485.3268	−1.03	C_30_H_46_O_5_	MS^2^[485]: 423.3287(100)		Ceanothic acid isomer	Other components
291.	33.54	485.3272	485.3268	−0.96	C_30_H_46_O_5_	MS^2^[485]: 423.3271(100)		Ceanothic acid isomer	Other components
292.	34.74	453.3010	453.3008	−0.62	C_29_H_42_O_4_	MS^2^[453]: 409.3102(100)		Ursonic acid	Other components
293.	36.81	545.3484	545.3481	−0.44	C_32_H_50_O_7_	MS^2^[545]: 499.3415(100)		Hovenidulcigenin B	Other components
294.	37.57	271.2273	271.2277	−0.55	C_16_H_32_O_3_	MS^2^[271]: 225.2217(100), 253.2169(3)		Hydroxypalmitic acid	Other components
295.	38.20^a^	455.3531	455.3526	0.66	C_30_H_48_O_3_	MS^2^[455]: 391.2346(100), 409.2462(56), 325.12858(53)		Oleanic acid	Other components

^a^ Identified by comparison with standards.

#### 3.3.1 Identification of flavonoid components

Compounds 77, 80, 212, 215, 219, 220, 223, 231, 237, 245, 258, 268, and 284 were eluted at 5.32, 5.51, 9.69, 9.27, 9.38, 9.57, 9.92, 10.50, 10.88 11.38, 11.38, 14.26, and 27.02 min, respectively, with the deprotonated molecular ion [M‐H]^−^ at *m/z* 577.1350 (C_30_H_25_O_12_, −0.24 ppm), 289.0715 (C_15_H_13_O_6_, −0.90 ppm), 609.1458 (C_27_H_29_O_16_, −0.59 ppm), 463.0880 (C_21_H_19_O_12_, −0.54 ppm), 463.0880 (C_21_H_19_O_12_, −0.34 ppm), 447.0928 (C_21_H_19_O_11_, −1.02 ppm), 593.1510 (C_27_H_29_O_15_, −0.33 ppm), 593.1512 (C_27_H_29_O_15_, −0.53 ppm), 447.0929 (C_21_H_19_O_11_, −0.86 ppm), 435.1292 (C_21_H_23_O_10_, −1.20 ppm), 435.1297 (C_21_H_23_O_10_, 0.86 ppm), 301.0350 (C_15_H_9_O_7_, −1.35 ppm), and 593.1297 (C_30_H_25_O_13_, −0.61 ppm), respectively; these were characterized as procyanidin B2, catechin, quercetin‐3‐O‐rutinoside, hyperoside, isoquercitrin, luteolin‐7‐O‐glucoside, kaempferol‐3‐O‐neohesperidoside, nicotiflorin, quercitrin, phlorizin, trilobatin, quercetin, and proanthocyanidins, respectively, by comparing the retention times and MS and MS^2^ information with those of the standards.

Compounds 76 and 134, which possessed the same quasi‐molecular ions and characteristic fragment ion as compound 80, were characterized as catechin isomer. Likewise, compounds 103, 135, 208, 211, 224, and 250 were the isomers of procyanidin B2, phlorizin, quercetin‐3‐O‐rutinoside, isoquercitrin, phlorizin, and luteolin‐7‐O‐glucoside, respectively.

Compounds 21 and 65, found at 2.12 and 4.91 min, respectively, possessed the same quasi‐molecular ion [M‐H]^−^ at *m/z* 435.0933 (C_20_H_19_O_11_) and their fragment ions [M‐H]^−^ at *m/z* 289.0716, 109.0282, and 125.0231, respectively; this indicates that they possessed the catechin moiety. Considering the neutral loss of xyluronic acid reside (C_5_H_6_O_5_, 146 Da), they were identified as catechin‐O‐xyluronate. Likewise, compounds 59 and 100 were catechin‐O‐glucoside (Ismail et al., 2019).

Compounds 180, 187, and 193, which appeared at a retention time of 8.02, 8.29, and 8.47 min, respectively, possessed the same quasi‐molecular ion [M‐H]^−^ at *m/z* 433.1140, and fragment ions at *m/z* 313.0717 ([M‐H‐120]^−^), 343.0827 ([M‐H‐90]^−^); they were identified as naringenin‐C‐glucosides (Karar & Kuhnert, 2015). Similarly, compound 115 was tentatively proposed as naringenin‐6,8‐di‐C‐glucoside (Uysal et al., 2021). Compound 202, found at 8.87 min, possessed the quasi‐molecular ion at [M‐H]^−^ *m/z* 433.1140 and the daughter ion at *m/z* 271.0609; it was attributed to the loss of a glucose moiety (C_6_H_10_O_5_, 162 Da) and tentatively identified as naringenin‐O‐glucoside (Abu‐Reidah et al., 2012).

Compounds 123, 130, 177, 178, and 190 were eluted at 6.61, 6.72, 7.79, 7.94, and 8.35 min, respectively, with the molecular formula C_33_H_40_O_21_ at *m/z* 771.1989. The fragment ion *m/z* 301.0352 was attributed to the loss of a glucose+rutinose group (C_18_H_30_O_14_, 470 Da), and further yielded the characteristic fragments ion at *m/z* 271.0248 and 151.0027, which indicate that they possessed quercetin moiety. Then, they were tentatively characterized as quercetin‐O‐glucoside‐rutinoside (Peng et al., 2018). Likewise, compounds 125, 183, 185, 196, 209, 213, 229, 236, and 243 were tentatively proposed as quercetin‐O‐lactoside, quercetin‐O‐rutinoside‐rhamnoside, quercetin‐O‐rutinoside‐arapaioside, quercetin‐O‐glucoside‐arabinoside, quercetin‐O‐glucuronide‐rhamnoside, quercetin‐O‐glucuronide‐rhamnoside, quercetin‐O‐arabinoside, quercetin‐O‐rhamnoside‐arabinoside, and quercetin‐O‐rhamnoside‐arabinoside, respectively (Abu‐Reidah et al., 2012; Peng et al., 2018; Zengin et al., 2019).

Compounds 233 and 238, eluted at 10.62 and 10.88 min, respectively, yielded a deprotonated ion [M‐H]^−^ *m/z* 623.1618 and the fragment ion at *m/z* 315.0505, corresponding to the neutral loss of rutinose moieties (C_12_H_20_O_9_, 308 Da); they were deduced as methylquercetin‐O‐rutinosides (Semple et al., 2016). Similarly, compound 214 was methylquercetin‐O‐rutinoside‐arabinoside.

Compounds 154, 163, 171, 173, 181, and 195, detected at 7.19, 7.31, 7.49, 7.64, 8.13, and 8.55 min, respectively, possessed the same quasi‐molecular ions [M‐H]^−^ at *m/z* 625.1410 and the main daughter ion at *m/z* 317.0299—which may be due to the loss of a rutinose group (C_12_H_20_O_9_, 308 Da)—and were identified as myricetin‐O‐rutinoside by referring to the literature (Peng et al., 2018).

Compounds 129 and 197 with the same quasi‐molecular ions at [M‐H]^−^ *m/z* 303.0510 were eluted at 6.71 and 8.65 min, respectively, and identified as taxifolin isomers according to the MS and MS/MS spectra (Tóth et al., 2015).

Compound 227 was detected at 10.34 min, possessing the quasi‐molecular ions [M‐H]^−^ at *m/z* 447.0933, the main MS/MS fragment ion at *m/z* 285.0402 formed by the neutral loss of glucose moiety (C_6_H_10_O_5_, 162 Da), which further produce the characteristic fragment ions at *m/z* 255.0292, 227.0341. Therefore, they were deduced as kaempferol‐O‐glucoside (Zengin et al., 2019). Likewise, compounds 126, 138, 143, 157, and 210 were considered kaempferol‐O‐rutinoside‐arabinoside‐glucoside, kaempferol‐O‐dirutinoside, kaempferol‐O‐rutinoside‐glucoside, kaempferol‐O‐rutinoside‐glucoside, and kaempferol‐O‐rutinoside‐arabinoside, respectively. Compound 132, observed at 6.79 min, possessed the quasi‐molecular ions at [M‐H]^−^ *m/z* 449.1089, and the main daughter ion at *m/z* 259.0607, 269.0451, 287.0554, and was tentatively proposed to be maesopsin 4‐O‐glucoside (Peng et al., 2018). Likewise, compounds 216, 255, and 271 were 3′,5′‐di‐C‐β‐d‐glucosylphloretin, baicalin, and wogonin‐O‐glucuronide, respectively (Chen et al., 2018). Compounds 203, 259, and 263 were tentatively inferred as isoliquiritin isomers (Chen et al., 2018).

#### 3.3.2 Identification of phenylpropanoids components

Compounds 101, 151, and 175 with the same deprotonated ions [M‐H]^−^ at *m/z* 179.0339 (C_9_H_8_O_4_) were eluted at 6.10, 7.15, and 7.77 min, respectively; compound 101 was accurately characterized as caffeic acid by comparison with the reference standard. Compounds 151 and 175 were caffeic acid isomers. Compounds 46, 52, 61, 70, 84, and 86 yielded the same quasi‐molecular ion [M‐H]^−^ at *m/z* 341.0878 and were eluted at 3.97, 4.40, 4.78, 5.05, 5.59, and 5.67 min, respectively. All these compounds showed the fragment ions at *m/z* 179.0338 [M‐H‐glu]^−^, 161.0232 [M‐H‐glu‐H_2_O]^−^, 135.0438 [M‐H‐glu‐CO_2_]^−^ and might be considered caffeic acid‐O‐glucoside (Uysal et al., 2021). Likewise, compounds 33, 41, 50, 56, 62, 73, 75, 78, 121, and 221 were tentatively characterized by the same fragment ions (*m/z* 179.0338) through the neutral loss of lactose group (C_12_H_20_O_10_, 324 Da), gentianose group (C_18_H_30_O_15_, 486 Da), arabinose+rhamnose group (C_11_H_18_O_8_, 278 Da), lactose+glucuronic acid group (C_18_H_28_O_16_, 500 Da).

Compound 266 might be considered caffeic acid ethyl ester (Celli et al., 2007). Compounds 261, 265, 270, 232, 241, 242, 244, 247, and 249 possessed the same characteristic fragment ions as caffeic acid and were tentatively proposed as caffeic acid derivatives.

Compound 192 was found at 8.47 min, possessing the quasi‐molecular ion [M‐H]^−^ at *m/z* 193.0506, and was unambiguously identified as ferulic acid by comparison with reference standards. Compound 113 possessed the same quasi‐molecular ion and mass spectrum data as those of compound 192 and was identified as ferulic acid isomer. Compounds 87, 90, 117, 139, and 161 were eluted at 5.70, 5.80, 6.52, 6.90, and 7.27 min, respectively, possessing the same quasi‐molecular ion [M‐H]^−^ at *m/z* 355.1035. The daughter ion at *m/z* 193.0496 was attributed to the loss of a glucose moiety (C_6_H_10_O_5_, 162 Da) and generated the characteristic fragment ion of ferulic acid at *m/z* 134.0360, 149.0595, 178.0261. Therefore, they were considered ferulic acids‐O‐glucoside (Abu‐Reidah et al., 2012). Likewise, compounds 71, 82, 88, and 97 were tentatively proposed as ferulic acid‐O‐lactosides, and compound 264 was considered to be ferulic acid‐O‐glucoside‐glucuronide. Compounds 48, 69, 260, and 269 were ferulic acid derivatives. Compounds 246, 252, and 257, showing [M‐H]^−^ ions at *m/z* 517.1351, were observed at 11.52, 12.42, and 12.83 min, respectively; they showed fragment ions at *m/z* 179.0339 and 193.0506, simultaneously yielded a neutral loss of glucose moiety (C_6_H_10_O_5_, 162 Da), and were characterized as caffeoyl ferulic acid‐O‐glucosides. Similarly, compounds 221 and 251 were identified as caffeoyl ferulic acid‐O‐lactoside and dicaffeoyl ferulic acid‐O‐rhamnoside, respectively.

Compounds 57, 66, 79, 85, 105, and 127 appeared at retention times of 4.70, 4.92, 5.49, 5.59, 6.23, and 6.69 min, respectively, possessing the same quasi‐molecular ions at [M‐H]^−^ *m/z* 325.0929. The main fragment ions *m/z* 163.0388 were generated based on the neutral loss of glucose reside (C_6_H_10_O_5_, 162 Da), and further produced the ion at *m/z* 119.0488, which was identified as coumaroyl hexose as reported in the literature (Zengin et al., 2019). Likewise, compounds 184, 222, 248, 262, and 267 were tentatively deduced as p‐Coumaric acid‐O‐glucoside‐arabinoside, p‐Coumaric acid‐O‐lactoside‐glucuronide, p‐Coumaric acid‐O‐lactoside‐glucuronide, p‐Coumaric acid‐O‐glucoside‐glucuronide, and p‐Coumaric acid‐O‐glucoside‐glucuronide, respectively. Compounds 33, 34, 43, 40, 53, 279, and 280 had the same characteristic fragment ion as that of p‐Coumaric acid and were identified as p‐Coumaric acid derivatives. Compounds 60, 67, and 147 were identified as dihydrocoumaroyl‐O‐glucosides (Hegazi et al., 2021) and compound 199 was identified as coumaroyl shikimate by matching its fragmentation pattern with those reported in literature (Zengin et al., 2019).

Compound 92, detected at 5.84 min, possessed the quasi‐molecular ions at [M‐H]^−^ *m/z* 177.0193 and was identified as esculetin (Tóth et al., 2015). Similarly, compounds 55 and 63 were deduced as esculin isomers (Tóth et al., 2015).

#### 3.3.3 Identification of alkaloid components

Compounds 94 and 172, eluted at 5.87 and 7.57 min, respectively, with the precursor ion at *m/z* 330.1700 and 342.1705, respectively, were characterized as sinomenine and magnoflorine by comparison with the standards.

Compounds 109 and 179, with the molecular formula C_19_H_24_NO_3_^+^, were found at 6.33 and 7.98 min, respectively; the molecular ions were obtained at *m/z* 314.1751 and the daughter ions were at *m/z* 269.1178 and 237.0913, respectively. These compounds were tentatively proposed as magnocurarine isomers (Shang et al., 2020). Compounds 107, 112, and 131 yielded a quasi‐molecular ion at *m/z* 356.1498, and were eluted at 6.26, 6.45, and 6.73 min, respectively. All these compounds showed the fragment ions at *m/z* 311.0921 and 285.1118 possibly due to the loss of C_2_H_7_N group (45 Da) and C_4_H_9_N group (71 Da), respectively; through data comparison with the literature, they might be considered (s)‐4‐keto‐magnoflorine isomers (Yan et al., 2013). Compounds 114 and 162, found at 6.47 and 7.29 min, respectively, yielded parent ions at *m/z* 344.1862 and were deduced as (s)‐tembetarine isomers according to the MS and MS/MS spectra (Yan et al., 2013). Compounds 119, 133, 137, 145, 160, and 166, observed at 6.54, 6.79, 6.86, 6.97, 7.26, and 7.43 min, respectively, had the same precursor ion at *m/z* 328.1543 and the fragment ion at *m/z* 283.0985 generated by the loss of the C_2_H_7_N residue (45 Da), which further gave rise to the product ions at *m/z* 265.0865 based on the neutral loss of H_2_O residue (18 Da). These compounds were tentatively proposed as desmethyl magnoflorine (Li et al., 2018). Similarly, compounds 128, 140, 152, and 170, detected at 6.70, 6.91, 7.15, and 7.48 min, respectively, possessed the same quasi‐molecular ions at [M + H]^+^ *m/z* 358.1654, fragment ion at *m/z* 313.1076, and 340.1543 generated by the loss of the C_2_H_7_N group (45 Da), H_2_O group (18 Da); they were identified as n‐methyllaudanidinium isomers (Yan et al., 2013). Compounds 226 and 230 had quasi‐molecular ions at *m/z* 296.1645 and 374.1598, yielded the fragment ions *m/z* 251.1069 and 329.1025, respectively, by the same loss of the C_2_H_7_N moieties (45 Da); they were deduced as nuciferine and chiloenamine, respectively (Yan et al., 2013). Likewise, compounds 93, 116, 120, 144, 158, 164, 167, 168, 169, 182, 186, 189, 191, 198, 207, and 239 were tentatively deduced based on previous reports (Shang et al., 2020; Tian et al., 2016; Xia et al., 2014).

Compound 136, detected at 6.82 min, possessed the quasi‐molecular ion [M + H]^+^ at *m/z* 286.1438 and the typical fragment ion *m/z* 269.1178 by the loss of the NH_3_ group (17 Da). Based on previously reported literature, it was deduced as coclaurine (Shang et al., 2020). Likewise, compounds 68, 72, and 122 were norcoclaurine isomers (Shang et al., 2020); compounds 81, 89, 188, and 201 were n‐methylcoclaurine isomers (Yan et al., 2013); compounds 74, 156, and 176 were reticulin isomers (Singh et al., 2016); compounds 104, 124, and 155 were norreticuline isomers (Menéndez‐Perdomo et al., 2021); and compounds 102 and 153 were hydroxylation products of coclaurine (Shang et al., 2020). Compounds 83 and 148, eluted at 5.54 and 7.07 min, respectively, had the molecular formula C_23_H_29_NO_8_ at *m/z* 448.1966 and the fragment ion *m/z* 286.1444 due to the neutral loss of the glucose reside (C_6_H_10_O_5_, 162 Da); they were identified as coclaurine‐O‐glucosides. Compound 150 was norcoclaurine‐O‐glucoside.

Compound 254, observed at 12.69 min, possessed the quasi‐molecular ion [M + H]^+^ at *m/z* 282.1489 and the fragment ions *m/z* 265.1229, 250.0994, and 219.0809; it was identified as O‐nornuciferine, consistent with the literature (Yan et al., 2013). Likewise, compounds 165, 206, and 225 were identified as juzirine, (r)‐asimilobine isomer, and (r)‐asimilobine isomer, respectively (Shang et al., 2020; Yan et al., 2013).

#### 3.3.4 Identification of organic acid components

Compound 4, possessing the deprotonated ion [M‐H]^−^ at *m/z* 191.0561 (C_7_H_12_O_6_), was detected at 0.89 min and characterized as quinic acid by comparing the retention time, MS, and MS^2^ information with the standards. Compounds 9 and 12, possessing the same quasi‐molecular ions and characteristic fragment ion as compound 4, were characterized as quinic acid isomers.

Compounds 14, 111, 174, and 218, eluted at 1.59, 6.41, 7.76, and 9.52 min, respectively, yielded the same parent ion [M‐H]^−^ *m/z* 151.0401 and were deduced as vanillin isomers (Tóth et al., 2015). Compounds 22, 23, and 159, with quasi‐molecular ions at [M‐H]^−^ *m/z* 167.0350, were eluted at 2.18, 2.27, and 7.24 min, respectively, and the typical daughter ions were obtained at *m/z* 152.0102 [M‐H‐CH_3_]^−^, 123.0437 [M‐H‐COOH]^−^, and 108.0203 [M‐H‐COOH‐CH_3_]^−^, respectively; they were identified as vanillic acid isomers (Tóth et al., 2015). Compounds 24 and 29, detected at 2.34 and 2.59 min, respectively, possessed the same precursor ion at [M‐H]^−^ *m/z* 329.0878 and fragment ions at *m/z* 167.0338, 152.0102, and 123.0437 and were identified as vanillic acid‐O‐glucosides (Abu‐Reidah et al., 2012). Likewise, compounds 99 and 108 were detected as vanillic acid‐O‐rhamnoside and vanillic acid‐O‐rutinoside, respectively (Zheleva‐Dimitrova et al., 2021). Compounds 18, 19, and 253 were vanillic acid derivatives, while compounds 35 and 36 were vanillin derivatives.

Compounds 44, 45, 205, and 217, with a molecular mass of *m/z* 137.0244, were found at 3.72, 3.86, 8.97, and 9.45 min, respectively; all these compounds yielded the fragment ions *m/z* 93.0331, which were consistent with 4‐hydroxybenzoic acid (Huang et al., 2019). Compounds 16, 27, 32, 37, 38, 51, and 54 were tentatively identified as 4‐hydroxybenzoic acid‐β‐glucoside (Peng et al., 2018); the main product ions at *m/z* 137.0244 were obtained by the loss of glucose residue (C_6_H_10_O_5_, 162 Da). Likewise, compound 96 was 4‐hydroxybenzoic acid‐O‐rutinoside. Compounds 17, 20, 28, and 30 were observed at 1.95, 2.10, 2.47, and 2.62 min, respectively, possessing the same quasi‐molecular ions [M‐H]^−^ at *m/z* 153.0193; they were identified as dihydroxybenzoic acid (Uysal et al., 2021). Compounds 15 and 25 possessed the same quasi‐molecular ions [M‐H]^−^ at *m/z* 315.0722, and the MS/MS fragment ions obtained at *m/z* 153.0188 indicated the loss of a glucose group (C_6_H_10_O_5_, 162 Da); these were tentatively identified as dihydroxybenzoic acid‐O‐glucosides.

Compounds 2 and 6 were eluted at 0.86 and 1.14 min, respectively, with a quasi‐molecular ion [M‐H]^−^ at *m/z* 191.0197 (C_6_H_8_O_7_) and the fragment ion at *m/z* 111.0073, suggesting that they were citric acid isomers (Zhang et al., 2020). Similarly, compounds 11 and 13 were tentatively assigned to 3‐methylglutaric acid isomers (Bruderer et al., 2017). Compounds 26, 31, 39, 42, 47, 49, 118, 146, 149, 194, and 256 were well characterized according to their MS and MS/MS spectra (Jung et al., 2019; Meng et al., 2020; Ostrowski et al., 2014; Ricciutelli et al., 2019; Tóth et al., 2015).

#### 3.3.5 Identification of other components

By comparing the retention time and fragment ions with reference compounds, Compounds 7, 8, and 295 generated a deprotonated molecular ion [M‐H]^−^ at *m/z* 344.0401 (C_10_H_11_O_7_N_5_P, −0.17 ppm), 328.0453 (C_10_H_11_O_6_N_5_P, 0.05 ppm), and 455.3526 (C_30_H_46_O_3_, 0.66 ppm), respectively, and were unambiguously characterized as CGMP, CAMP, and OA, respectively.

Compound 10, eluted at 1.37 min, exhibited a quasi‐molecular ion at [M‐H]^−^ *m/z* 490.0981, generated the main fragment ion at *m/z* 328.0432 by the loss of a glucose group (C_6_H_10_O_5_, 162 Da), and was identified as CAMP‐O‐glucoside. Compounds 1, 3, and 5, found at 0.81, 0.89, and 1.08 min, respectively, possessed the same quasi‐molecular ion at [M‐H]^−^ *m/z* 341.1089 and were tentatively identified as sucrose isomers based on a previous study (Zhang et al., 2020).

### 3.4 Pharmacological activity of the *Z. jujuba*

Various constituents, including phenylpropanoids, flavonoids, alkaloids, organic acids, and other components, were discovered in *Z. jujuba*. Among them, phenylpropanoid compounds such as ferulic and caffeic acid have attracted wide attention in improving diabetes and treating cardiovascular and cerebrovascular diseases (Chao et al., 2010). Moreover, the flavonoid compounds, including procyanidin B2, proanthocyanidins, and quercitrin, have anti‐inflammation, antioxidant, and antibacterial activity (Ivanišová et al., 2017). Hence, an increasing number of people like to drink water soaked in *Z. jujuba* as it improves immunity and delays aging.

As nitrogen‐containing substances of natural origin and of limited distribution, alkaloids possess a complex structure and show significant pharmacological activity. For example, sinomenine and magnoflorine possess anti‐inflammatory, analgesic, antitumor, and other biological active properties, and can be used to treat acute and rheumatoid arthritis (Qian et al., 2007). This study is the first to report alkaloid compounds in *Z. jujuba*. The result is helpful for the development of *Z. jujuba* in food, medical, and pharmaceutical industries.

## 4 CONCLUSION

In the present study, UHPLC‐Q‐Exactive Orbitrap MS combined with PRM has been confirmed to be a powerful analytical technique for the separation and detection of chemical compounds in *Z. jujuba*. In this study, 295 compounds including 69 flavonoids, 60 alkaloids, 82 phenylpropanoids, 52 organic acids, and 32 other compounds were tentatively identified via the accurate mass determination of the quasi‐molecular ion and MS^2^ fragmentation pattern, and of these, 270 have been reported in *Z. jujuba* for the first time. To our knowledge, this is the first report describing the comprehensive characterization of the chemical constituents of *Z. jujuba*.

## CONFLICT OF INTEREST

All contributing authors have no conflicts of interest to declare.
